# Molecular Phylogeny of the Leafy Liverwort *Lejeunea* (Porellales): Evidence for a Neotropical Origin, Uneven Distribution of Sexual Systems and Insufficient Taxonomy

**DOI:** 10.1371/journal.pone.0082547

**Published:** 2013-12-18

**Authors:** Jochen Heinrichs, Shanshan Dong, Alfons Schäfer-Verwimp, Tamás Pócs, Kathrin Feldberg, Aleksandra Czumaj, Alexander R. Schmidt, Joachim Reitner, Matt A. M. Renner, Joern Hentschel, Michael Stech, Harald Schneider

**Affiliations:** 1 Systematic Botany and Mycology, Faculty of Biology, Ludwig Maximilian University, Munich, Germany; 2 Department of Systematic Botany, Albrecht von Haller Institute of Plant Sciences, Georg August University, Göttingen, Germany; 3 Mittlere Letten 11, Herdwangen-Schönach, Germany; 4 Botany Department, Institute of Biology, Eszterházy College, Eger, Hungary; 5 Courant Research Centre Geobiology, Georg August University, Göttingen, Germany; 6 Royal Botanic Gardens & Domain Trust, Sydney, Australia; 7 Department of Systematic Botany with Herbarium Haussknecht and Botanical Garden, Friedrich Schiller University, Jena, Germany; 8 Naturalis Biodiversity Center, Leiden, The Netherlands; 9 Leiden University, Leiden, The Netherlands; 10 The Natural History Museum London, London, United Kingdom; Field Museum of Natural History, United States of America

## Abstract

**Background:**

*Lejeunea* is a largely epiphytic, subcosmopolitan liverwort genus with a complex taxonomic history. Species circumscriptions and their relationships are subject to controversy; biogeographic history and diversification through time are largely unknown.

**Methodology and Results:**

We employed sequences of two chloroplast regions (*trn*L-*trn*F, *rbc*L) and the nuclear ribosomal ITS region of 332 accessions to explore the phylogeny of the *Harpalejeunea*-*Lejeunea*-*Microlejeunea* complex. *Lejeunea* forms a well-supported clade that splits into two main lineages corresponding to *L.* subg. *Lejeunea* and *L.* subg. *Crossotolejeunea*. Neotropical accessions dominate early diverging lineages of both main clades of *Lejeunea*. This pattern suggests an origin in the Neotropics followed by several colonizations from the Neotropics into the Paleotropics and vice versa. Most Afro-Madagascan clades are related to Asian clades. Several temperate *Lejeunea* radiations were detected. Eighty two of the 91 investigated *Lejeunea* species could be identified to species level. Of these 82 species, 54 were represented by multiple accessions (25 para- or polyphyletic, 29 monophyletic). Twenty nine of the 36 investigated species of *L.* subg. *Lejeunea* were monoicous and 7 dioicous. Within *L.* subg. *Crossotolejeunea*, 15 of the 46 investigated species were monoicous and 31 dioicous. Some dioicous as well as some monoicous species have disjunct ranges.

**Conclusions/Significance:**

We present the first global phylogeny of *Lejeunea* and the first example of a Neotropical origin of a Pantropical liverwort genus. Furthermore, we provide evidence for the Neotropics as a cradle of *Lejeunea* lineages and detect post-colonization radiations in Asia, Australasia, Afro-Madagascar and Europe. Dioicy/monoicy shifts are likely non-randomly distributed. The presented phylogeny points to the need of integrative taxonomical studies to clarify many *Lejeunea* binomials. Most importantly, it provides a framework for future studies on the diversification of this lineage in space and time, especially in the context of sexual systems in Lejeuneaceae.

## Introduction

The taxonomic history of the leafy liverwort *Lejeunea* Lib. is best characterized as a story of controversial opinions on species delimitation and assumed relationships. Libert [1] described the genus based on only two species, *Lejeunea calcarea* Lib. [nowadays treated as *Cololejeunea calcarea* (Lib.) Schiffn.] and *Lejeunea serpyllifolia* Lib., the latter being a synonym of *Lejeunea cavifolia* (Ehrh.) Lindb. [2], [3]. Soon after Libert's publication, the genus became widely recognized and numerous new species were described [4]. Until the end of the 19th century, the number of *Lejeunea* species exceeded one thousand [5] but early authors applied a wider genus concept than is accepted today. A good example in this regard is the treatment of Spruce [6] who classified *Lejeunea* in 39 subgenera. The majority of these subgenera was elevated to genus rank by Schiffner [7]. Subsequently, further new genera were introduced consisting of former *Lejeunea* species (e.g., [8]–[10]). As a consequence, *Lejeunea* species sensu Spruce [6] were placed in some 60 different genera [11].

Recent taxonomic and/or molecular phylogenetic studies of Lejeuneaceae led to a considerable reduction of genera [12]–[18]. This trend becomes particularly apparent in *Lejeunea* since more than a dozen generic names were recently lowered to synonyms of this genus [15], [18], [19]. *Lejeunea* is currently circumscribed by long-inserted leaves, divided or undivided underleaves, leaf lobules with an unreduced first tooth and a marginal hyaline papilla, small, segmented or homogeneous oil bodies, lack of ocelli, lejeuneoid innovations, unwinged female bracts and inflated perianths with 0–5 smooth or toothed wings [17], [20]. *Lejeunea* is recognized for its morphological disparity. Diversification time estimates indicated an origin of *Lejeunea* in the early Cenozoic [21]–[23]. The genus has its centre of diversity in the humid tropics where the species usually grow as epiphytes on stems, branches, twigs and leaves of a large number of cormophytes but also on rock [24]. Although the vast number of species occur exclusively in tropical climates, the genus is also well represented in temperate regions with a humid climate [25], [26].

According to recent estimates the species diversity of *Lejeunea* may exceed three hundred [27], however, the precise number of species is still unclear due to the limited availability of modern revisionary studies [13], [28]–[30]. According to current knowledge, *Lejeunea* includes narrow endemics [29] as well as intercontinentally distributed species such as the subcosmopolitan *Lejeunea flava* (Sw.) Nees [31]. Intercontinental ranges have been accepted for many liverwort species due to an extensive morphological overlap of remote populations and the production of spores and propagules suitable for long-distance dispersal [32]–[34], although molecular phylogenetic studies incorporating multiple accessions of morphologically-typologically circumscribed liverwort species usually demonstrate a considerable genetic variation and a structure that is related to spatial ranges rather than to morphological disparities [35]–[40]. These studies also demonstrated the para- or polyphyly of many morphologically circumscribed liverwort species [36], [41], [42].

The objective of this study is to reconstruct the first comprehensive phylogeny of *Lejeunea* using chloroplast and nuclear DNA markers. This phylogenetic framework is used to reconstruct the origin of the genus and infer evidence, which supports dispersal between the Neotropics and the Paleotropics [40], respectively the hypothesis of a tropical origin of the extant temperate species diversity [22], [43]. In addition, we infer the evolution of reproductive systems with the focus on monoicy and dioicy in the evolution of *Lejeunea*. Finally, we test current morphological-typological species concepts by including multiple accessions and examine whether the recovered phylogenetic relationships correspond to/or conflict with morphologically circumscribed taxa.

## Results

### Phylogeny - Reduced dataset

**Figure 1:**
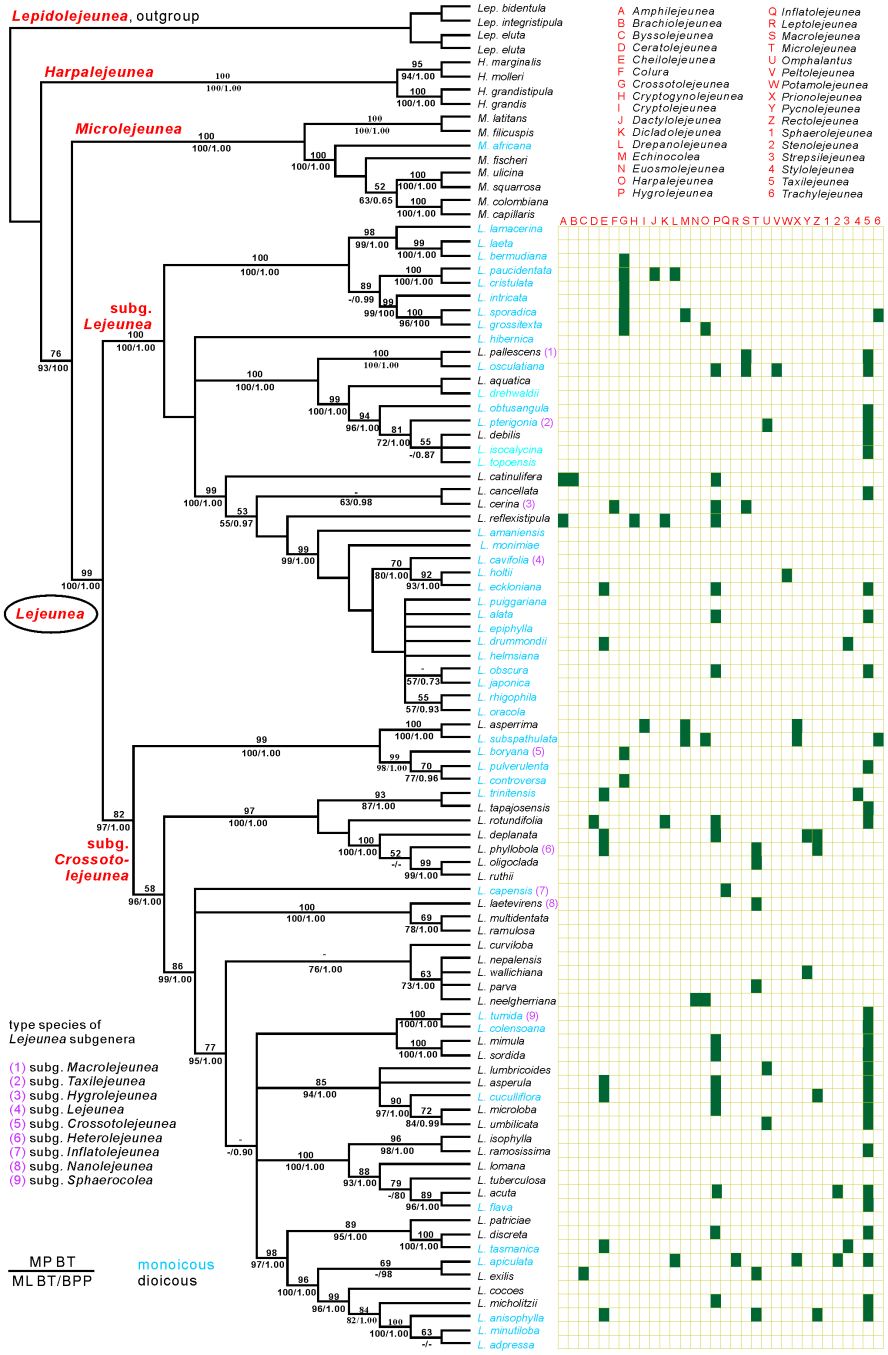
Strict consensus of 4578 equally parsimonious trees derived from the reduced dataset. MP and ML bootstrap percentage values and Bayesian Posterior Probabilities are indicated at branches. Monoicous species are given in blue, dioicous species in black. Type species of subgenera of *Lejeunea* are marked and alternative genus assignments of *Lejeunea* species shown.

The reduced dataset comprised one accession per ingroup species (specimens identified only to genus level excluded). Of a total of 2,351 character sites, 725 were parsimony informative, 248 unique to a single accession and 1,378 constant. The maximum parsimony (MP) analysis resulted in 4,578 most parsimonious trees with the features: length of 4,016 steps, consistency index of 0.38, and retention index of 0.69 (Figure 1). Bayesian inference of phylogeny and maximum likelihood (ML) analyses recovered consensus trees respectively optimal trees that were highly similar in their topologies to each other as well as to the MP tree. The four representatives of *Harpalejeunea* (Spruce) Schiffn. formed a clade that was placed sister to a clade comprising two clades of which one included eight *Microlejeunea* Steph. species whereas the other one was composed by 82 *Lejeunea* species. The monophyly of *Lejeunea* achieves bootstrap percentage values (BPVs) of 99 or 100% and a Bayesian Posterior Probability (BPP) of 1.00 (Figure 1). The *Lejeunea* clade consisted of two main lineages corresponding to *Lejeunea* subg. *Lejeunea* (BPV MP 100%, ML 100%, BPP p = 1.00) and *L.* subg. *Crossotolejeunea* Spruce (BPV MP 82% ML 97%, BPP p = 1.00). In past classification, the investigated *Lejeunea* species were alternatively placed in 32 different genera, with up to 6 different treatments per species (Figure 1). *Lejeunea* species previously treated as *Taxilejeunea* (Spruce) Schiffn. were diffusely distributed and nested in most *Lejeunea* clades. Elements of *Crossotolejeunea* (Spruce) Schiffn. were found in both main lineages of *Lejeunea*. Twenty nine of the 36 investigated representatives of *Lejeunea* subg. *Lejeunea* were monoicous (81%) and 7 (19%) dioicous (Figure 1). Within *Lejeunea* subg. *Crossotolejeunea*, 15 of the 46 investigated species were monoicous (33%) and 31 dioicous (67%). Ancestral character reconstruction recovered dioicy as the likely ancestral state of *Lepidolejeunea* R.M.Schust., *Harpalejeunea*, and *Microlejeunea*, whereas the ancestral state of *Lejeunea* was found to be equivocal in maximum parsimony reconstructions. In maximum likelihood reconstructions, dioicy was found to be ancestral with a probability of 0.75 versus a probability of 0.25 for monoicy. The ancestral state of *L.* subg. *Lejeunea* was either resolved as equivocal (50% of most parsimonious trees) or monoicous (50% of most parsimonious trees). ML reconstructions recovered a probability of monoicy of 0.71. Similarly, the ancestral state of subg. *Crossotolejeunea* was found to be equivocal in all most parsimonious trees but showed a probability of 0.77 to be dioicous (Table 1).

**Table 1:** Ancestral character reconstruction of dioicous/monoicous reproductive systems.

	MP Dioicy	MP Monoicy	ML Dioicy	ML Monoicy
*Lepidolejeunea*	yes	no	0.93	0.07
*Harpalejeunea*	yes	no	0.98	0.02
*Microlejeunea*	yes	no	0.93	0.07
*Lejeunea*	equivocal	equivocal	0.75	0.75
*L.* subg. *Lejeunea*	equivocal (50%), no (50%)	equivocal (50%), yes (50%)	0.29	0.71
L. subg. *Crossotolejeunea*	equivocal	equivocal	0.77	0.23
clade *L. lamacerina*-*L. grossitexta*	no	yes	0.01	0.99
clade *L. hibernica*-*L. oracola*	no	yes	0.29	0.71
clade *L. pallescens*- *L. topoensis*	no	yes	0.24	0.76
clade *L. catinulifera*-*L. oracola*	yes	no	0.75	0.25
clade *L. amaniensis*-*L. oracola*	no	yes	0.10	0.90
clade *L. asperrima*-*L. controversa*	equivocal	equivocal	0.40	0.60
clade *L. trinitensis*-*L. adpressa*	yes	no	0.92	0.08
clade *L. trinitensis*-*L. ruthii*	yes	no	0.96	0.04
clade *L. capensis*-*L. adpressa*	yes	no	0.97	0.03

The reconstruction is based on the reduced dataset using Maximum Parsimony (MP) and Maximum Likelihood (ML).

### Phylogeny - Large dataset

The large dataset consisted of 2,351 character sites (909 parsimony informative, 1,212 constant). The MP analysis resulted in more than 350,000 equally parsimonious trees with a length of 6,427 steps, a consistency index of 0.30 and a retention index of 0.83 (not depicted).

**Figure 2:**
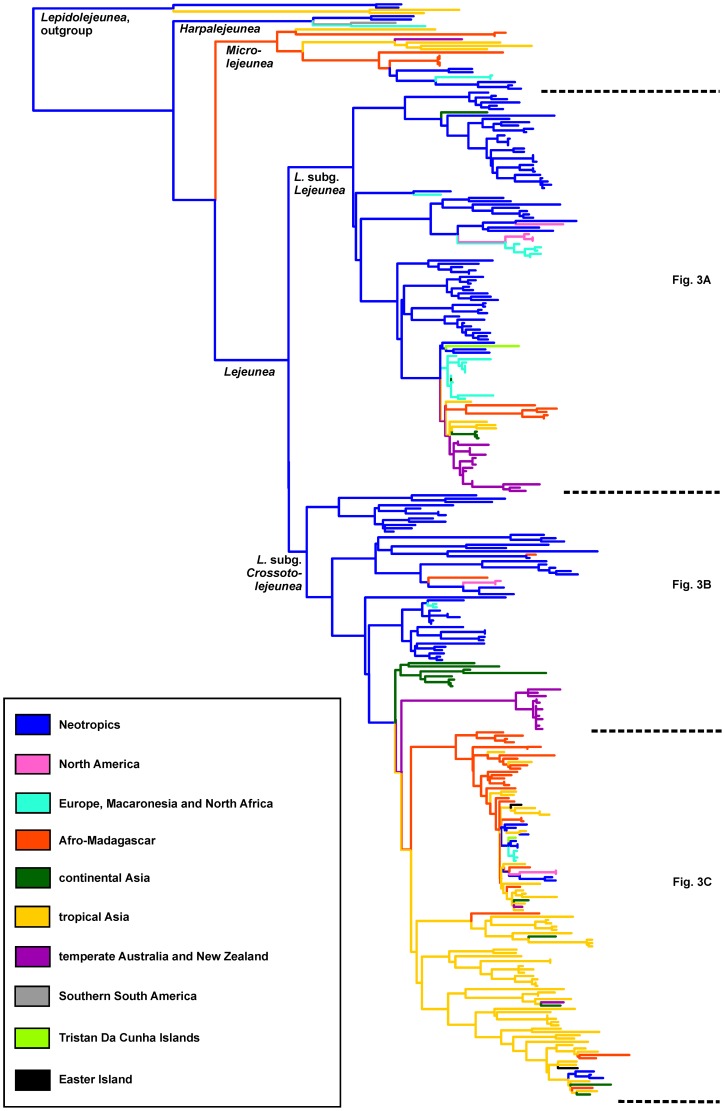
Condensed Maximum Likelihood phylogeny of the *Harpalejeunea*-*Lejeunea*-*Microlejeunea* clade. Branch colors correspond to the most parsimonious reconstruction of ancestral areas of distribution and provide evidence for a Neotropical origin of *Lejeunea*.

**Figure 3:**
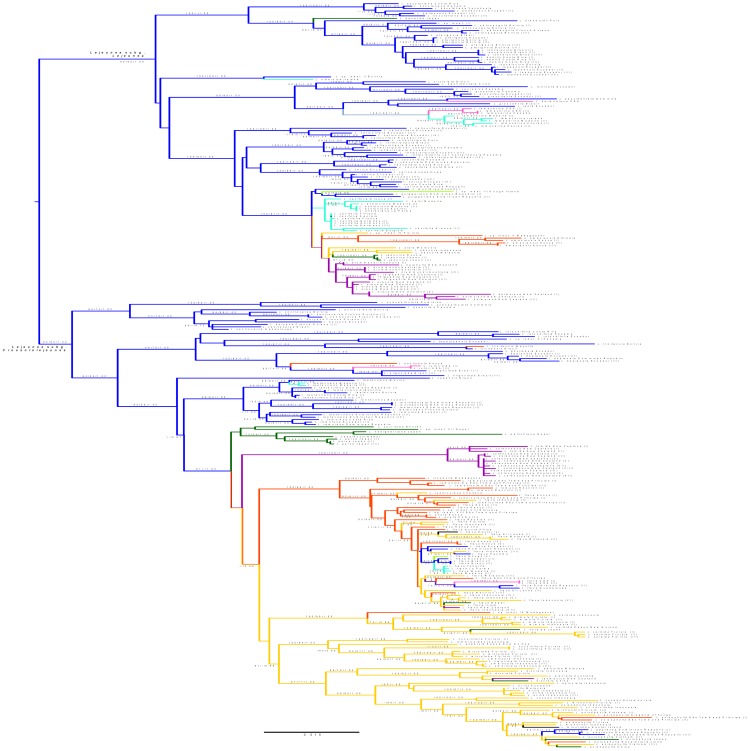
Pruned *Lejeunea* clade from Figures 2/S1. ML and MP bootstrap percentage values as well as Bayesian Posterior Probabilities are indicated at branches. Fifty four *Lejeunea* species are represented by multiple accessions, 29 of these are monophyletic, 25 para- or polyphyletic.

The ML phylogeny based on the large dataset is shown in Figure S1. A condensed version without species labeling is depicted in Figure 2. The *Lejeunea* clade was pruned and split in three parts, which are depicted in Figure 3, with BPPs and ML/MP BPVs indicated at branches. The phylogeny was consistent to the topology derived from the reduced dataset albeit without good ML BPV for *Lejeunea* subg. *Crossotolejeunea* (ML BPV = 65%). Out of the 82 *Lejeunea* species with reliable species identification, 54 were represented by multiple accessions. Twenty five of these 54 *Lejeunea* species were resolved as para- or polyphyletic, whereas 29 were monophyletic. Intercontinental ranges of several *Lejeunea* species were confirmed.

### Biogeography

**Figure 4:**
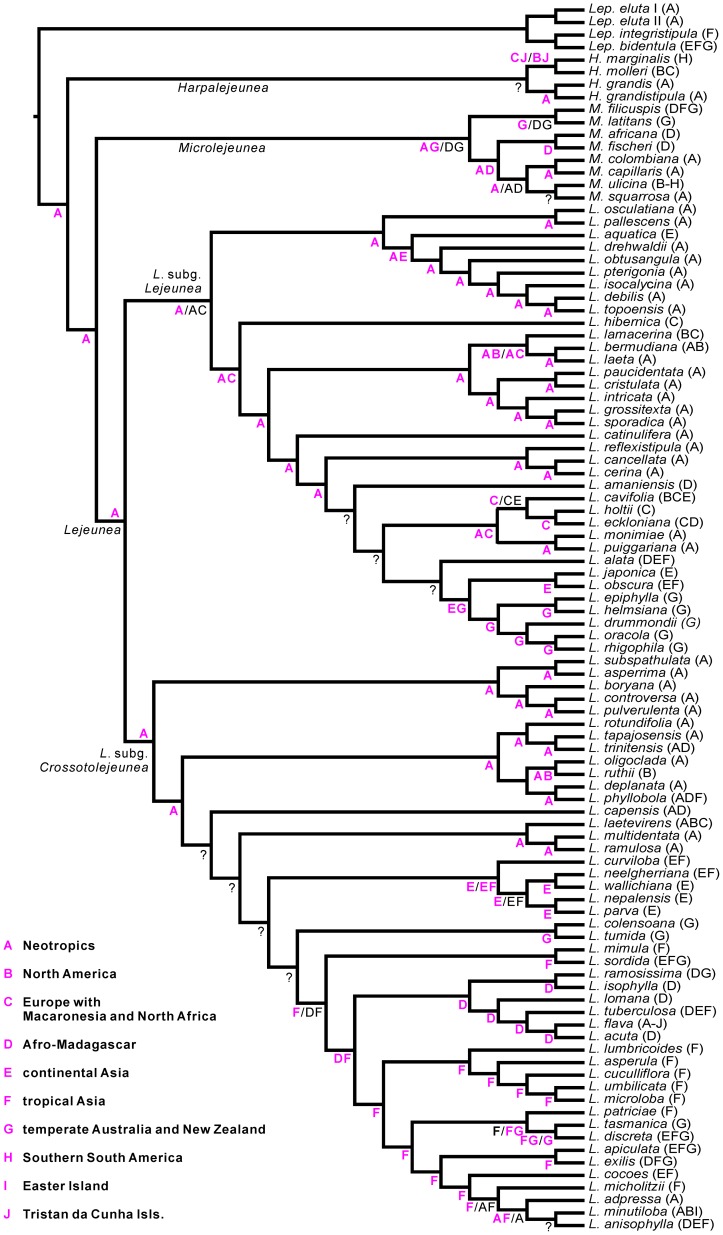
Ancestral areas of distribution reconstructed using S-DIVA. The distribution of each species is given in brackets according to the ancestral areas of distribution scheme. Putative ancestral areas of distribution are shown at nodes, in case of alternative results the less likely solution is given in black. Question marks indicate ambiguities [more than two alternative proposals]. The reconstruction points to a Neotropical origin of *Lejeunea*.

The most parsimonious reconstruction of ancestral areas of distribution based on the large dataset (Figures 2, 3 A–C) indicated a Neotropical origin of *Lejeunea* as well as of its subgenera *Crossotolejeunea* and *Lejeunea*. The S-Diva reconstruction generated from the reduced dataset suggested two scenarios. In scenario one both subgenera originated in the Neotropics, whereas in the other scenario two alternative solutions were found for *L.* subg. *Crossotolejeunea* (Figure 4). In the second scenario, *L.* subg. *Crossotolejeunea* originated in an area comprising the Neotropics but also Europe plus Macaronesia and North Africa. African and Asian accessions were found to be nested in derived lineages. *Lejeunea* subg. *Crossotolejeunea* comprised a species rich radiation in Afro-Madagascar, Africa, and Asia that likely originated from a single colonization of the Paleotropics from the Neotropics. Each four clades of *Lejeunea* were recovered with occurrences in Australasia or North America respectively, five clades with occurrences in Macaronesia and Atlantic Europe, and seven clades with occurrences in temperate/subtropical Asia (Figure 3). The subcosmopolitan *L. flava* complex nested in an African lineage. Accessions from Gough Island were resolved in Neotropical lineages; accessions from Easter Island in tropical Asian clades. The African-Neotropical *L. trinitensis* Lindenb. & Gottsche nested in a Neotropical clade; the Neotropical *L. adpressa* Nees in a clade dominated by Asian accessions. North American accessions of *L. lamacerina* (Steph.) Schiffn. are placed sister to European/Macaronesian accessions.

## Discussion

### Supraspecific classification

Recent molecular phylogenetic studies identified a monophylum with representatives of *Harpalejeunea*, *Microlejeunea* and *Lejeunea* [17]. Furthermore, a recent report showed that the putatively allied genus *Bromeliophila* R.M.Schust. [20] forms a sister relationship with *Prionolejeunea* (Spruce) Schiffn. rather than nesting in *Lejeunea* [44]. Morphologically, *Lejeunea* differs from the former two genera by a lack of ocelli [the sole representative of *Lejeunea* with ocelli, *L. huctumalcensis* Lindenb. & Gottsche, belongs to another main lineage of Lejeuneaceae (Czumay et al., unpublished)]. The monophyly of *Harpalejeunea*, *Microlejeunea* and *Lejeunea* is confirmed in our study, with *Microlejeunea* placed sister to *Lejeunea*.

*Lejeunea* has been classified in some 50 subgenera of which 17 are still accepted as part of *Lejeunea*. These subgenera are usually defined by one or a few morphological character states, and their recognition and circumscription is subject to controversy. A good example is *Lejeunea* subg. *Taxilejeunea* which was alternatively treated as separate genus *Taxilejeunea*, and as such accepted by several recent authors [9], [12], [45], [46], although the morphology of both genera is largely overlapping [12]. This situation is reflected in our phylogeny, with *Taxilejeunea* elements in nearly all lineages of *Lejeunea* (Figure 1). The problematic circumscription of *Lejeunea* taxa is also reflected in the alternative placement of the 82 identified species of our study in 32 different genera of Lejeuneaceae (Figure 1), with one species in up to six genera (*Lejeunea apiculata* Sande Lac.). *Lejeunea* splits into two main clades with heterogeneous morphology. One includes the generitype *L. serpyllifolia* ( = *L. cavifolia*) and the types of three further subgenera; the other clade comprises types of four different subgenera, the oldest available subgenus name being *L.* subg. *Crossotolejeunea* Spruce (type: *L. boryana* Mont.) (Figure 1). *Lejeunea* subg. *Crossotolejeunea* was proposed for monoicous species with decurved and acuminate leaf apices, and 5-keeled perianths with denticulate and fimbriate keels [6]. A few years later, *Crossotolejeunea* was raised to generic rank [7]. However, *Crossotolejeunea* was synonymized with *Lejeunea* because the diagnostic character combinations were found to be inconsistent among species considered to belong to *Crossotolejeunea* [13]. The polyphyly of *Crossotolejeunea* as circumscribed by Spruce [6] is confirmed in the presented study by recovering *Crossotolejeunea* representatives in both main clades of *Lejeunea* (Figure 1). However, the presence of the type species *L. boryana* in the second main clade allows the assignment of *L.* subg. *Crossotolejeunea*. Incongruence of morphology-based classifications and molecular phylogenies was reported for a rapidly increasing number of genera of liverworts such as *Athalamia* Falc. [47], *Cololejeunea* (Spruce) Schiffn. [48], *Diplasiolejeunea* (Spruce) Schiffn. [40], *Frullania* Raddi [49], *Plagiochila* (Dumort.) Dumort. [50], *Porella* L. [51], *Radula* Dumort. [52], *Scapania* (Dumort.) Dumort. [53], *Syzygiella* Spruce [54], and *Telaranea* Schiffn. [55]. Together, these studies clarified the phylogeny of these liverworts and provided the foundation to introduce new classifications using holophyly as the main criterion [40], [52], [56]–[58]. Unfortunately, many of these newly circumscribed taxa lack obvious morphological diagnostic characters hampering assignments of species to these clades using solely morphology.

In this study we propose to assign the two main *Lejeunea* clades to *Lejeunea* subg. *Crossotolejeunea* and *Lejeunea* subg. *Lejeunea* but hesitate to establish further supraspecific entities. In our opinion, it is premature to introduce a comprehensive classification of the two subgenera into sections since our *Lejeunea* sampling is still rather incomplete in the context of taxonomic sampling. In addition, further studies are required to explore the morphological features of species recovered in well supported clades. A good example in this regard is the morphological treatment of *L. pulverulenta* (Gottsche ex Steph.) M.E.Reiner [46]. In this study, *L. pulverulenta* was assumed to be aligned with *L. controversa* Gottsche and *L. cerina* (Lehm. & Lindenb.) Gottsche et al. based on morphological similarities, e.g. the papillose leaf cells with trigones and intermediate cell wall thickenings. A sister relationship of *L. pulverulenta* and *L. controversa* (*L.* subg. *Crossotolejeunea*) is confirmed (Figure 1) but *L. cerina* is found to be nested in *Lejeunea* subg. *Lejeunea* instead of *L.* subg. *Crossotolejeunea*.

The morphology of many *Lejeunea* species has not yet been exhaustively studied and our knowledge is often restricted to descriptions of the gross morphology of the gametophyte. Schuster [9], [31] repeatedly pointed to the taxonomical value of character states visible only in living plants, namely the oil bodies, and the sporophytes. Only recently it was shown that the rough surface of *Lejeunea* species is not necessarily caused by papillae but can also result from the production of surface waxes [59]. We need comprehensive morphological datasets of gametophytes and sporophytes besides expansion of molecular datasets to establish a hierarchical classification of *Lejeunea* into subgenera and sections. These data will also demonstrate whether clades share certain morphologies or can only be defined by DNA sequence evidence.

### Circumscription of species

The present study addressed the reliability of current morphological-typological species concepts in *Lejeunea* by sampling multiple accessions of several currently accepted species. In the absence of studies on speciation processes and the maintenance of species borders in *Lejeunea*, we consider three criteria - diagnostic morphology, biogeographic consistency, and reciprocal monophyly - as the most reliable procedure to identify putative species [60]. Congruence between the phylogenies derived from either the nuclear or the chloroplast markers is interpreted as evidence for reproductive isolation. Hence we regard incongruence of morphologically circumscribed taxa with molecular phylogenies as evidence for the limitations of our current morphology-based classification. However, integration of molecular and exhaustive morphological data allows often but not always for a reconsideration of morphological features considered to be of diagnostic importance and result in modified species circumscriptions (e.g., [61]–[64]). These short-term solutions are practical and helpful despite the amount of efforts required. In addition, they may allow to recognize the extent of the failure of current taxonomic practice.

Multiple accessions of 29 *Lejeunea* species formed monophyletic lineages but 25 species proved to be para- or polyphyletic (Figure 3 A–C). The ratio of nearly 50% rejection of currently accepted species is remarkable and requires further using of more comprehensive datasets and analyses. These datasets may expand not only the number of accessions studied per species but also explore the genetic diversity by employing markers that will allow a more comprehensive study of the genotypic distinction such as ISSRs, AFLPs, and SNPs. Exhaustive studies with such marker-systems hold special promises for lineages with a low clade diversity such as the *Lejeunea cavifolia* – *L. eckloniana* Lindenb. – *L. holtii* Spruce-complex. The high number of non-monophyletic *Lejeunea*-species indicates that our current morphology-based classification does not adequately consider the possible presence of morphologically cryptic or semicryptic entities, and local endemism [38], [62], [65]. Some studies reported evidence for rather limited morphological variation among *Lejeunea* species and thus morphologically similar plants may be placed in different main clades of *Lejeunea*. A good example is the *Lejeunea tumida* Mitt. complex whose representatives are placed in both main clades of *Lejeunea* although they were earlier treated as a single species [30], [42]. This observation is consistent with the results available for other genera of Lejeuneaceae, namely *Marchesinia* Gray [36], *Ptychanthus* Nees [66], *Mastigolejeunea* (Spruce) Schiffn. and *Thysananthus* Lindenb. [67]. All these studies suggest that we currently underestimate Lejeuneaceae species diversity. Examples supporting this notion are reported here with *Lejeunea flava* and *L. laetevirens* Nees & Mont., which may in fact represent complexes including several independent entities. *Lejeunea flava* has been studied exhaustively using morphological evidence and several subspecies or segregates have been proposed [10], [31], [68], [69]. However, we were not able to adopt these taxonomical concepts for our phylogeny (Figure 3 C) although we could recognize some morphological tendencies and found the morphologically well separated species *L. acuta* Mitt. and *L. tuberculosa* Steph. nested in the *L. flava* clade. The *L. laetevirens* complex is similarly problematic since our phylogeny indicates that several still unrecognized entities hide in *L. laetevirens* s.l.: A robust clade with Neotropical and Macaronesian accessions of *L. laetevirens* is placed sister to a Neotropical clade with *L. laetevirens* morphotypes as well as multiple accessions of *L. multidentata* M.E.Reiner & Mustelier and *L. ramulosa* (Herzog) R.M.Schust. The latter two species differ from *L. laetevirens* by dentate or acute leaves. *Lejeunea multidentata* was aligned with *L. boryana* Mont. and *L. controversa* rather than with *L. laetevirens* based on shared dull appearance caused by strongly papillose cells [70], [71], however, according to our phylogenies these species are not closely related. An extension of the sampling is necessary to revise the taxonomy of the *L. laetevirens* clade. The same holds true for the polyphyletic *L. anisophylla* Nees & Mont. and several other problematic binomials.

### Dispersal biogeography

Liverworts produce spores and small propagules that are capable for distribution through air currents over larger distances [72], [73]. However, population studies of liverworts generally show a spatial distribution of genetic diversity that does not correspond to a general panmixis hypothesis [74], [75]. Thus, the current distribution of liverworts is not random and biogeographic studies frequently recover conserved biogeographic patterns that can be interpreted by considering the combination of processes such as occasional long distance dispersal, frequent dispersal over short distances, local extinction, and local diversification [76], [77]. The reported distribution of *Lejeunea* suggests that this genus is not an exception and that conserved spatial patterns exist. Although the limited availability of lejeuneoid fossils prevents us from a detailed reconstruction of divergence times (the two Miocene fossils *Lejeunea* sp. [78] and *Lejeunea palaeomexicana* Grolle [79] cannot be assigned to any of our *Lejeunea* clades) an early Cenozoic origin of the genus can be assumed based on the existing estimates [21]–[23]. This time frame provides information about the position of the continents which is important in distinguishing between establishment via long-distance dispersal versus vicariance as the preferred explanation for the observed disjunct ranges. Dispersal over larger distances seems to occur only infrequently in *Lejeunea*, as is indicated by the clear geographical structure of disjunct species as well as multi-species clades. A good example is the *L. lamacerina* clade that splits into a North American and a European/Macaronesian lineage, without any evidence of recent geneflow. The unsatisfactory taxonomy of many other investigated clades hampers similar statements, however, the long branches in many morphologically circumscribed species and their para- or polyphyly provide evidence for local diversification/speciation. Evidence for lacking or restricted geneflow between distant liverwort populations has been demonstrated several times [74], [80] and can also be concluded for *Lejeunea*. Local diversification subsequent to successful long-distance dispersal seems to dominate the evolutionary history of *Lejeunea*. Accordingly, the majority of the investigated *Lejeunea* species has regional distribution ranges but about 23% of the identified species are more widespread and occur in at least two of our ten putative areas of endemism. Examples include the Neotropical-Macaronesian range of *L. laetevirens*, the Neotropical-Asian range of *L. trinitensis* Lindenb. & Gottsche (Figure 3 B) and the African-Asian range of *L. anisophylla* (Figure 3 C).

### Neotropical origin

The early diverging lineages of both main clades of *Lejeunea* occur predominantly in the Neotropics. Thus, our reconstructions revealed a Neotropical origin of *Lejeunea* with subsequent dispersal into other tropical as well as temperate regions. A Neotropical origin has been shown for several lineages of angiosperms, namely Burmanniaceae [81], Burseraceae [82], Gentianaceae [83] and Malpighiaceae [84]. It has also been discussed for the grammitid clades of polygrammoid ferns [85], [86] and the Neotropical-African liverwort *Bryopteris* (Nees) Lindenb. [87] but has not yet been proposed for any subcosmopolitan liverwort genus based on molecular data. This is partly caused by the limited access to comprehensive phylogenies of species-rich liverwort genera [40], [49], [51]–[54], [76], [77], [88]. The lejeuneoid genus *Diplasiolejeunea* shows a somewhat different pattern with a deep split into a Paleotropical and a Neotropical clade [40], but a few Pantropical species soften this otherwise strict separation by indicating occasional intercontinental dispersal events. In contrast to the pattern in *Diplasiolejeunea* both main clades of *Lejeunea* show a more even representation of putative regions of endemism, indicating that long distance dispersal is more frequent in *Lejeunea* than in *Diplasiolejeunea* as long as we assume similar ages for both genera.

Our topologies point to several dispersal events from the Neotropics into Africa (*L. trinitensis*, *L. phyllobola* Nees & Mont.). This pattern is not uncommon in leafy liverworts and has been recovered for *Herbertus juniperoideus* (Sw.) Grolle [77], *Marchesinia brachiata* (Sw.) Schiffn. [36], *Plagiochila boryana* Steph. [56] and the genus *Bryopteris* [87]. The subcosmopolitan *L. flava* complex appears to have originated in Africa and subsequently colonized large parts of the tropics and adjacent regions, with several dispersal events between the Old and the New World. This pattern of older spatial separations followed by young inter-continental dispersals was reported for a few plants such as the fern genus *Nephrolepis* Schott [89] and the pantropical liverwort *Plagiochila* sect. *Vagae* Lindenb. [56]. Our phylogenies support close relationships of African and Asian *Lejeunea* floras, however, the Neotropical *L. adpressa* is of Paleotropical, most likely Asian, origin (Figure 3 C). *Lejeunea*-accessions from the Polynesian Easter Island are likewise related to Asian clades whereas the *Lejeunea* accessions from Gough Island (Southern Atlantic Ocean) are nested in Neotropical lineages. A South American origin of Gough Island liverworts has already been demonstrated for the genus *Herbertus* [90]. The Macaronesian accessions of *L. laetevirens* are nested in a Neotropical clade, indicating dispersal from the Neotropics into Macaronesia. This pattern seems to be common in leafy liverworts and has also been reconstructed for species of *Plagiochila* [91] and *Leptoscyphus* Mitt. [92].

### The tropics as a cradle and museum

*Lejeunea* has its centre of diversity in the humid lowlands and lower montane sites of the tropics; its diversity in temperate regions is considerably lower. This pattern is consistent with the widely recognized latitudinal biodiversity gradient [43], [93]–[96]. Various hypotheses have been introduced to explain the origin of this gradient (see [43] for review) of which some involve the rather controversial concept of niche conservatism. So far, very little attention has been given to latitudinal biodiversity gradients in seed-free land plants, but is starting to be explored in ferns (see [97]) and here in the liverwort genus *Lejeunea*. In accordance with the general hypothesis of a latitudinal diversity gradient, *Lejeunea* includes only a few temperate lineages, which are in each case nested in tropical clades.

The pattern observed for *Lejeunea* appears to be consistent with the role of the tropics as a cradle and museum of diversity [98]–[100], and mirrors observations for the whole family Lejeuneaceae [22]. Liverwort families with a centre of diversity in the tropical highlands can show considerably different patterns and may have entered the tropics from temperate regions [76]. Interestingly, temperate species were not always found to possess a tropical sister species but evidence for several radiations in temperate regions were discovered, including two multi-species clades with occurrences in Australasia, one with occurrences in temperate Asia, and two with occurrences in Macaronesia and Atlantic Europe (Figs 3 A–C). The discovery of these clades provides opportunities to test some of the arguments concerning the origin of the latitudinal diversity gradient such as niche conservatism and different speciation rates [97], [101]. The recovery of radiations in the temperate climate zones of Australasia resembles the recent report of a New Zealand radiation of grammitid ferns [102]. Grammitid ferns share with *Lejeunea* their origin in tropical regions and their preference to climates with high humidity. These examples may indicate the possibility of high speciation rates in temperate climates caused by ecological opportunities. The observed change in the climatic niche preferences is again consistent with reports in tree ferns growing in the wet temperate climates of Australasia [103].

### Sexual systems in a largely epiphytic genus

About two third of liverworts are dioicous [104] whereupon the distribution of dioicous and monoicous species differs from genus to genus. The speciose genus *Plagiochila* is a prime example of a completely dioicous group whereas monoicous species dominate in *Cololejeunea* (Spruce) Schiffn., *Riccia* L. and *Riccardia* Gray [24], [105]. The evolution of sexual systems has so far been studied for only two genera of liverworts using a phylogenetic framework: the largely epiphytic leafy liverworts *Radula* Dumort. and *Diplasiolejeunea* [40], [106]. Only 16 of the ca. 200 *Radula* species are monoicous whereas monoicy and dioicy is more evenly distributed in *Diplasiolejeunea*. Single monoicous species of *Radula* were resolved in several otherwise dioicous clades, a similar supposedly random pattern was observed in *Diplasiolejeunea*. Monoicy in *Radula* was also not correlated with obligate epiphytism but occurred in facultative epiphytic lineages [106].

In *Lejeunea* we observed an uneven distribution of sexual systems (Figure 1). *Lejeunea* subg. *Lejeunea* is dominated by monoicous species whereas dioicous species dominate in *L.* subg. *Crossotolejeunea*. Similarly to the situation in *Radula*, some monoicous species clustered in clades dominated by dioicous species, in particular in *L.* subg. *Crossotolejeunea*. However, monoicous species are the most frequent in *L.* subg. *Lejeunea* and our character reconstruction (Table 1) recovered some indications for the transition from dioicy to monoicy in the early diversification of the genus. We also found evidence for a rather frequent change of the reproductive system during the history of the genus with a minimum number of character state changes: five times in *L.* subg. *Lejeunea* and nine times in *L.* subg. *Crossotolejeunea*.

Monoicous species are potentially capable to produce sporophytes through self-fertilization. On one hand this may allow a more frequent establishment of new populations via long distance dispersal, but on the other hand this may result in invariable genotypes, accumulation of genetic load, and limited adaptation to new environments [105]. However, dioicy is not necessarily a barrier to regular sporophyte development. Many *Plagiochila* species frequently produce sporophytes as do at least some dioicous species of *Frullania* and *Porella* [50], [105]. Thus, future studies need to explore the accumulation of genetic load, effective population size, and the temporal stability of habitats as factors that shape the evolution of reproductive systems in *Lejeunea*.

According to existing data, both dioicous and monoicous *Lejeunea* species are able to form disjunct ranges. However, disjunctions over large distances might not necessarily be the result of spore dispersal but could also be caused by vegetative reproduction through propagules. Vegetative reproduction plays an important role in the range formation of liverworts and enhances the chances of establishing in a new environment, especially for dioicous species. A dioicous long-distance disperser is trapped in a very small area unless it is able to colonize its new environment through vegetative distribution. Accordingly the likelihood of the arrival of spores of the other sex clearly increases with range expansion through vegetative distribution. However, *Lejeunea* includes only few species that frequently produce propagules [107], despite wide species distribution ranges. A further aspect may be variation in the extinction risks caused by the different sexual systems but very little evidence exists to evaluate this factor.

Schuster [31] emphasizes the importance of monoicy for species colonizing unstable epiphytic habitats but many *Lejeunea* species are dioicous. This trend is even more evident in the sister genus *Microlejeunea* which is nearly completely dioicous [24], despite its general preference for epiphytic habitats. The same applies to *Radula*. Devos et al. [106] speculate that dioicous epiphytes often distribute vegetatively, not only through specialised propagules but also through unspecialized gametophyte fragments, and that they are often not strictly depending on epiphytic environments. Kraichak [108] reinforces this argument by demonstrating a correlation of reproduction through asexual propagules and an epiphyllous mode of life in Lejeuneaceae.

Currently the importance of monoicy for an epiphytic mode of life and long distance dispersal is rather unclear since the available studies point to more complex interrelationships. Future studies should not only focus on an extension of the phylogenetic sampling and improvements of the underlying taxonomy but also on the ecological ranges of disjunct liverworts. Intercontinentally distributed *Diplasiolejeunea* species have broader ecological amplitudes compared to geographically more restricted species [40], allowing for the colonization of a larger number of environments and enhancing the chance of a permanent establishment. We also need comprehensive studies on the resistance of spores and vegetative propagules of liverworts against drought and frost and the ability of sporophyte production under suboptimal climatic conditions.

### Perspectives

*Lejeunea* is a prime example to illustrate the current state of affairs in liverwort classification. After three centuries of morphology-based research a plethora of taxa have been proposed in this genus, of which only a small part has been included in modern revisions, reflecting the limited number of liverwort specialists dealing with these taxonomically difficult plants. Our molecular data add to growing evidence that not all biologically relevant entities can be detected using solely morphology, and that the acceptance of a considerable intraspecific morphological variation may lead to an underestimation of the actual number of biological species [109], [110]. Thus, concepts considering cryptic and semi-cryptic species may provide more realistic estimates than the current practice. Based on our topology it is possible to identify species complexes that are not yet properly understood and that need to be studied using extended datasets. We urgently need molecular studies incorporating numerous accessions of morphologically circumscribed species from throughout their range. Only combined molecular-morphological studies will allow to understand range formation and to establish more natural species circumscriptions [111]. These studies will also facilitate estimates of the real number of biological species of liverworts. It is not unlikely that a portion of these species will not exhibit morphological disparities or can at best been identified using statistical methods and larger series of reference specimens [112]. In such a situation, reference sequences ( = DNA barcodes) are the most promising approach to obtain reliable identifications of these plants [113]. However, the establishment of the DNA barcodes needs to go hand-in-hand with critical taxonomic revision of species-rich genera like *Lejeunea*. The reported phylogeny provides the framework enabling the design and management of these studies because the major task of taxonomic revisions can be separated in groups of species belonging to the same clade.

## Materials and Methods

### Taxon sampling and outgroup selection

Taxa studied, including GenBank accession numbers and voucher details, are listed in Table S1. Ingroup taxa were selected according to availability and to represent the morphological variation and geographical distribution of *Lejeunea*. Representatives of the sister genera *Harpalejeunea* and *Microlejeunea* [17] were included to test the *Lejeunea* genus concept. Multiple accessions of several species were used to explore intraspecific genetic variation. Representatives of *Lepidolejeunea* were selected as outgroup species based on the analyses of [14] and [17]. Altogether 332 accessions from the herbaria AK, DUKE, EGR, GOET, JE, L, or NSW were used for this study.

### DNA extraction, PCR amplification and sequencing

Upper parts of a few gametophytes were isolated from herbarium specimens. Total genomic DNA was extracted using Invisorb Spin Plant Mini Kit (Invitek, Berlin, Germany) prior to amplification. Protocols for PCR were carried out as described in previous publications: *rbc*L gene and *trn*L-F region from [114], and nrITS1-5.8S-ITS-2 region from [87]. Bidirectional sequences were generated using a MegaBACE 1000 automated sequencing machine using BigDye® Terminator v3.1 Cycle Sequencing Kit (Applied Biosystems, Foster City, CA, USA). Sequencing primers were those used for PCR. Newly generated sequences were assembled and edited using SeqAssem [115]. Seven hundred and nineteen sequences were newly generated for this study; 175 sequences were downloaded from Genbank.

### Phylogenetic analyses

All sequences were aligned manually in Bioedit version 7.0.5.2 [116] resulting in a *rbc*L alignment with 895 positions, *trn*L-F 441 and an nrITS alignment with 1,015 putatively homologous sites. Ambiguous positions were excluded from all alignments and lacking data were coded as missing. Two datasets were compiled and analysed separately: dataset 1 ( = large dataset) included all studied accessions, whereas dataset 2 ( = reduced dataset) included only one accession per identified ingroup species. Accessions identified only to genus level were excluded from dataset 2. Phylogenetic trees based on the reduced dataset were used to visualize the current supraspecific classification of *Lejeunea* and to explore the evolution of monoicy/dioicy.

Maximum parsimony (MP) analyses were carried out with PAUP* version 4.0b10 [117]. MP heuristic searches of the comprehensive and the reduced datasets were conducted with the following options implemented: heuristic search mode, 1,000 random-addition-sequence replicates, tree bisection-reconnection (TBR) branch swapping, MULTrees option on, and collapse zero-length branches off. All characters were treated as equally weighted and unordered. Non-parametric bootstrapping values [118] were generated as heuristic searches with 1,000 replicates, each with ten random-addition replicates. The number of rearrangements was restricted to ten millions per replicate. Bootstrap percentage values (BPV)≥70 were regarded as good support [119]. Where more than one most parsimonious tree was found, trees were summarized as strict consensus tree(s). The three genomic regions and the combined chloroplast DNA dataset vs nrITS dataset were first analysed separately to check for topological incongruence. The consensus trees of the non-parametric bootstrap analyses were compared by eye to identify conflicting nodes supported by at least 70% [120]. The trees gave no evidence of incongruence. Accordingly, the datasets were combined.

The program jModeltest 0.1.1 [121] was used to select a best-fit model of sequence evolution for the maximum likelihood (ML) analyses of the each genomic region, using the Akaike information criterion. The following models were chosen for the respective data divisions: (*rbc*L) TPM1uf+I+G; (*trn*L-F) TVM+G and (nrITS) TIM3+G. A partitioned ML bootstrap analysis was conducted using the program Garli 2.0 [122]. The analysis was run until five million generations were completed without significant improvement (ln *L* increase of 0.01) to the topology. Node support was evaluated through 200 bootstrap replicates in which each repetition terminated after 100,000 generations were completed without topological improvements.

Bayesian inference was implemented in the program MrBayes 3.2 [123] allowing different models for each partition. Bayesian searches were carried out with four simultaneous Markov chains, ten million generations, and sampling every 1000^th^ generation. The first 25% of trees were discarded as burn-in. Bayesian posterior probability (BPP) confidence values were generated from trees saved after this initial burn-in. Values were regarded as significant when BPP≥0.95 [124].

### Ancestral areas of distribution

Data on distribution ranges of the investigated taxa were obtained from the literature. Given the wide distribution ranges of some species, the putative distribution range of endemism was coded as covering ten possible areas: Neotropics, North America, Southern South America, Europe with North Atlantic Islands (e.g. Macaronesia) and North Africa (Africa north of the Sahara), Afro-Madagascar (sub-Saharan Africa, Madagascar, Mascarenes, Seychelles, and São Tomé), continental Asia (comprising temperate and subtropical regions), tropical Asia (including Melanesia and tropical Australia), temperate Australia and New Zealand, Tristan da Cunha Islands and Easter Island. Ancestral areas of distribution were reconstructed using two different approaches. The first approach was based on the large dataset and considers the presence of several unidentified species with unclear distribution ranges. To overcome this problem, the putative region of endemism ( = the ten regions mentioned above, see also Fig. 2) of every accession was coded rather than the species range. Subsequently we reconstructed ancestral areas of distribution using MP criteria as implemented in Mesquite ver. 2.75 [125] based on the ML topology.

In the second approach we used dataset 2 including each one accession per identified species and a coding of the complete species range. Ancestral areas of distribution were reconstructed using S-DIVA [126] as implemented in RASP 2.0 based on 7,500 Bayesian trees from the reduced dataset.

### Evolution of reproductive systems

The occurrence of dioicous/monoicous reproductive systems was scored by evaluating the information provided in the literature for each species included in dataset 2 [13], [28], [31], [45], [68], [127]–[137]. In case both character states were indicated (*L. hibernica* Grolle, [131]), the species was scored as monoicous. These efforts resulted into a matrix of two character states without any polymorphic or unknown character states. To explore the evolution of this character, we used the results of the MP analyses of the reduced dataset. Maximum parsimony character reconstructions were carried out using Mesquite 2.75. The character states were plotted over all most parsimonious trees recovered in the MP analysis of the reduced dataset. Nodes absent from some of these trees were ignored. In addition, we carried out maximum likelihood analyses using the MK model [138] and the strict consensus tree obtained from the most parsimonious tree set.

## Supporting Information

Figure S1 Maximum Likelihood phylogeny of the *Harpalejeunea*-*Lejeunea*-*Microlejeunea* clade. ML and MP bootstrap percentage values as well as Bayesian Posterior Probabilities are indicated at branches. Branch colors correspond to the most parsimonious reconstruction of ancestral areas of distribution (see Figure 2).
(TIF)

Table S1 Taxa used in the present study. Information about the origin of the studied material, vouchers, as well as GenBank accession numbers is included. New sequences in bold face.
(DOC)
