# Supplementary material for: Molecular Phylogeny of the Leafy Liverwort Lejeunea (Porellales): Evidence for a Neotropical Origin, Uneven Distribution of Sexual Systems and Insufficient Taxonomy
Source: PLoS One. 2013 Dec 18;8(12):e82547. doi: 10.1371/journal.pone.0082547 (PMC3867362; doi:10.1371/journal.pone.0082547)
Supplement: Table S1 — Taxa used in the present study. Information about the origin of the studied material, vouchers, as well as GenBank accession numbers is included. New sequences in bold face. (DOC) [file pone.0082547.s002.doc]

**Table S1. Taxa used in the present study.** Information about the origin of the studied material, vouchers, as well as GenBank accession numbers is included. New sequences in bold face.

| *Taxon* | *Origin* | *Collector* | *GenBank Accession No.* | | |
| --- | --- | --- | --- | --- | --- |
|  |  |  | rbcL | trnL-F | nrITS |
| *Harpalejeunea. grandis* Grolle & M.E.Reiner | Colombia | Cleef 6450 (GOET) | KC313144 | KC313184 | KC313106 |
| *H. grandistipula* R.M.Schust. | Ecuador | Schäfer-Verwimp et al. 24163/B (GOET) | KC313145 | KC313185 | KC313107 |
| *H. marginalis* (Hook.f. & Taylor) Steph. | Chile | Schäfer-Verwimp & Verwimp 8082 (GOET) | KC313147 | KC313187 | KC313109 |
| *H. molleri* (Steph.) Grolle | Azores | Schäfer-Verwimp & Verwimp 29334 (GOET) | KC313148 | KC313188 | KC313110 |
| *Lejeunea acuta* Mitt. | Comoros | Pócs et al. 9276/CX (EGR) | **KF556383** | **KF556133** | **KF556601** |
| *L. acuta* | Kenya | Chuah-Petiot Mb 22 (JE) | **KF556384** | **KF556134** | **KF555917** |
| *L. adpressa* Nees | Dominican Rep. (I) | Schäfer-Verwimp & Verwimp 26931/B (GOET) | **KF556386** | **KF556136** | **KF555919** |
| *L. adpressa* | Dominican Rep. (II) | Schäfer-Verwimp & Verwimp 27215/F (GOET) | **KF556387** | **KF556137** | **KF555920** |
| *L. adpressa* | Dominican Rep. (III) | Pócs & Pócs 03156/F (EGR) | **KF556385** | **KF556135** | **KF555918** |
| *L. alata* Gottsche | Malaysia | Schäfer-Verwimp & Verwimp 18912 (GOET) | ----------- | **KF556140** | **KF555922** |
| *L. amaniensis* E.W.Jones | Ethiopia | Hylander 4589 (EGR) | **KF556393** | **KF556144** | **KF555925** |
| *L. amaniensis* | Kenya (I) | Malombe 5012/cau.x2 (EGR) | **KF556390** | **KF556141** | **KF555923** |
| *L. amaniensis* | Kenya (II) | Malombe & Chituyi 5006/Si.1aS5 (EGR) | **KF556392** | **KF556143** | **KF556603** |
| *L. amaniensis* | Kenya (III) | Malombe & Soita 5008/K.1cS5 (EGR) | **KF556391** | **KF556142** | **KF555924** |
| *L. anisophylla* Nees & Mont. | China | Koponen et al. 56008 (H) | AY125943 | AY144484 | AY125348 |
| *L. anisophylla* | Fiji Isls. | Pócs & Pócs 03307/O (EGR) | **KF556397** | **KF556149** | **KF555929** |
| *L. anisophylla* | Indonesia | Schäfer-Verwimp & Verwimp 21131 (GOET) | **KF556398** | **KF556150** | **KF555930** |
| *L. anisophylla* | Japan | Yamaguchi 24461 (GOET) | **KF556400** | **KF556152** | **KF555931** |
| *L. anisophylla* | Kingdom of Tonga | Schäfer 18341 (GOET) | **KF556394** | **KF556145** | **KF555926** |
| *L. anisophylla* | Equatorial Guinea | Müller B312/B (EGR) | **KF556399** | **KF556151** | **KF556605** |
| *L. anisophylla* | São Tomé and Príncipe (I) | Shevock & Daniel 34368 (EGR) | **KF556401** | **KF556153** | **KF556606** |
| *L. anisophylla* | São Tomé and Príncipe (II) | Pócs & Pócs 34749 (EGR) | **KF556395** | **KF556146** | **KF555927** |
| *L. anisophylla* | Vietnam (I) | Pócs 02103/V (EGR) | **KF556396** | **KF556148** | **KF556604** |
| *L. anisophylla* | Vietnam (II) | Pócs 02105/J (EGR) | **KF556529** | **KF556303** | **KF556629** |
| *L. anisophylla* | Vietnam (III) | Pócs 02103/C (EGR) | ----------- | **KF556147** | **KF555928** |
| *L. apiculata* Sande Lac. | Indonesia | Schäfer-Verwimp & Verwimp 20985 (GOET) | ----------- | **KF556154** | **KF555932** |
| *L. aquatica* Horik. | Japan | Higuchi 1021 (JE) | ----------- | **KF556155** | **KF555933** |
| *L. asperrima* Spruce | Panama | Schäfer-Verwimp & Verwimp 30817 (GOET) | **KF556402** | **KF556157** | **KF555935** |
| *L. asperula* (Steph.) Mizut. | Papua New Guinea | Streimann 40815 (JE) | ----------- | **KF556156** | **KF555934** |
| *L. bermudiana* (A.Evans) R.M.Schust. | USA | Shaw 14939 (DUKE) | **KF556403** | **KF556158** | **KF555936** |
| *L. boryana* Mont. | Ecuador | Schäfer-Verwimp & Nebel 33218 (GOET) | **KF556404** | ----------- | **KF555937** |
| *L. boryana* | French Guiana (I) | Hartmann et al. 04-054 (GOET) | ----------- | **KF556160** | ----------- |
| *L. boryana* | French Guiana (II) | Holz FG 00-0103 (GOET) | **KF556405** | **KF556159** | **KF555938** |
| *L. cancellata* Nees & Mont. | Costa Rica (I) | Schäfer-Verwimp & Holz SV/H-0507/C (GOET) | **KF556409** | **KF556164** | **KF555942** |
| *L. cancellata* | Costa Rica (II) | Schäfer-Verwimp & Holz SV/H-0374/A (GOET) | **KF556410** | **KF556165** | **KF556607** |
| *L. cancellata* | Dominican Rep. | Schäfer-Verwimp & Verwimp 27005 (GOET) | **KF556411** | **KF556166** | **KF556608** |
| *L. cancellata* | Panama | Schäfer-Verwimp & Verwimp 30850 (GOET) | **KF556408** | **KF556163** | **KF555941** |
| *L. capensis* Gottsche | Brazil | Schäfer-Verwimp & Verwimp 15057 (GOET) | ----------- | **KF556167** | **KF555943** |
| *L. catinulifera* Spruce | Ecuador | Gradstein & Mandl 10141 (GOET) | DQ983688 | DQ987411 | DQ987307 |
| *L. catinulifera* | Ecuador | Wilson et al. 04-01 (GOET) | DQ983687 | DQ987432 | DQ987328 |
| *L. catinulifera* | Ecuador | Schäfer-Verwimp et al. 24259/A (GOET) | **KF556413** | ----------- | **KF555945** |
| *L. catinulifera* | Ecuador | Schäfer-Verwimp et al. 24188 (GOET) | **KF556412** | **KF556168** | **KF555944** |
| *L. catinulifera* | Ecuador | Schäfer-Verwimp et al. 24248 (GOET) | **KF556414** | **KF556169** | **KF555946** |
| *L. cavifolia* (Ehrh.) Lindb. | Belgium | Heinrichs et al. 3816 (GOET) | **KF556419** | **KF556174** | **KF555950** |
| *L. cavifolia* | Finland | Ahonen s.n. (H) | AY125945 | AY144486 | AY125347 |
| *L. cavifolia* | France | Schäfer-Verwimp & Verwimp 27247 (GOET) | **KF556421** | **KF556176** | **KF555952** |
| *L. cavifolia* | Georgia | Hentschel Bryo 04382 (JE) | **KF556418** | **KF556173** | **KF555949** |
| *L. cavifolia* | Germany (I) | Heinrichs 3695 (GOET) | AY548102 | DQ238581 | DQ987259 |
| *L. cavifolia* | Germany (II) | Schäfer-Verwimp & Verwimp 28787 (GOET) | **KF556420** | **KF556175** | **KF555951** |
| *L. cavifolia* | Germany (III) | Schäfer-Verwimp & Verwimp 28806 (GOET) | **KF556415** | **KF556170** | **KF555947** |
| *L. cavifolia* | Greece (I) | Düll 1./10.5.2004 (JE) | **KF556417** | **KF556172** | **KF555948** |
| *L. cavifolia* | Greece (II) | Schäfer-Verwimp & Verwimp 15888 (GOET) | **KF556416** | **KF556171** | ----------- |
| *L. cerina* (Lehm. & Lindenb.) Gottsche, Lindenb. & Nees | Costa Rica | Schäfer-Verwimp & Holz SV/H-0471 (GOET) | **KF556425** | **KF556180** | **KF555955** |
| *L. cerina* | Dominican Rep. | Schäfer-Verwimp & Verwimp 26978 (GOET) | ----------- | **KF556182** | **KF555957** |
| *L. cerina* | Ecuador (I) | Schäfer-Verwimp & Nebel 32122 (GOET) | **KF556423** | **KF556178** | **KF555954** |
| *L. cerina* | Ecuador (II) | Wilson et al 04-02 (GOET) | DQ983686 | DQ987433 | DQ987329 |
| *L. cerina* | Ecuador (III) | Wilson et al 04-13 (GOET) | DQ983689 | DQ987441 | DQ987339 |
| *L. cerina* | Ecuador (IV) | Schäfer-Verwimp & Nebel 31942 (GOET) | **KF556422** | **KF556177** | **KF555953** |
| *L. cerina* | Ecuador (V) | Nöske & Holz 165 (GOET) | **KF556424** | **KF556179** | **KF556609** |
| *L. cerina* | Guadeloupe | Schäfer-Verwimp & Verwimp 22473 (GOET) | **KF556426** | **KF556181** | **KF555956** |
| *L. cocoes* Mitt. | Indonesia | Schäfer-Verwimp & Verwimp 21050 (GOET) | **KF556430** | **KF556186** | **KF555961** |
| *L. cocoes* | Solomon Isls. | Seaward 108088 (JE) | ----------- | **KF556187** | **KF555962** |
| *L. colensoana* (Steph.) M.A.M.Renner | New Zealand (I) | Renner 300101 (AK) | ----------- | JF308572 | JF308543 |
| *L. colensoana* | New Zealand (II) | Renner 300127a (AK) | ----------- | JF308576 | JF308547 |
| *L. colensoana* | New Zealand (III) | Renner 300044 (AK) | ----------- | JF308565 | JF308536 |
| *L. colensoana* | New Zealand (IV) | Renner 300130 (AK) | ----------- | JF308577 | JF308548 |
| *L. colensoana* | New Zealand (V) | Renner 300028 (AK) | ----------- | JF308562 | JF308533 |
| *L. colensoana* | New Zealand (VI) | Renner 300140 (AK) | ----------- | JF308578 | JF308549 |
| *L. colensoana* | New Zealand (VII) | Renner 300104 (AK) | ----------- | JF308573 | JF308544 |
| *L. colensoana* | New Zealand (VIII) | Renner 300103 (AK) | ----------- | JF308574 | JF308545 |
| *L. colensoana* | New Zealand (IX) | Renner 300127 (AK) | ----------- | JF308575 | JF308546 |
| *L. colensoana* | New Zealand (X) | Renner 300039 (AK) | ----------- | JF308564 | JF308535 |
| *L. colensoana* | New Zealand (XI) | Renner 300030 (AK) | ----------- | JF308563 | JF308534 |
| *L. controversa* Gottsche | Bolivia | Gradstein 7189 (JE) | ----------- | **KF556190** | **KF556610** |
| *L. controversa* | Brazil | Vital 8794 (GOET) | **KF556431** | **KF556188** | **KF555963** |
| *L. controversa* | French Guiana (I) | Hartmann et al. 04-033 (GOET) | **KF556432** | **KF556189** | **KF555964** |
| *L. controversa* | French Guiana (II) | Hartmann et al. 04-092 (GOET) | **KF556434** | **KF556192** | **KF555965** |
| *L. controversa* | Guadeloupe | Schäfer-Verwimp & Verwimp 22196 (GOET) | **KF556433** | **KF556191** | ----------- |
| *L. cristulata* (Steph.) M.E.Reiner & Goda | Brazil | Giancotti 17 (JE) | ----------- | **KF556193** | **KF555966** |
| *L. cuculliflora* (Steph.) Mizut. | Fiji Isls. (I) | Pócs & Pócs 03286/L (EGR) | ----------- | **KF556359** | **KF556110** |
| *L. cuculliflora* | Fiji Isls. (II) | Pócs & Pócs 03286/D (EGR) | **KF556435** | **KF556194** | **KF556611** |
| *L. cuculliflora* | Fiji Isls. (III) | Pócs & Pócs 03274/BL (EGR) | **KF556577** | **KF556358** | **KF556109** |
| *L. curviloba* Steph. | Bhutan | Long 10611 (JE) | ----------- | **KF556195** | **KF555967** |
| *L.* cf. *curviloba* | Indonesia | Gradstein 10300 (GOET) | **KF556427** | **KF556183** | **KF555958** |
| *L. debilis* (Lehm. & Lindenb.) Nees & Mont. | Costa Rica (I) | Dauphin 1793 (GOET) | **KF556436** | **KF556196** | **KF555968** |
| *L. debilis* | Costa Rica (II) | Biasi et al. 21 (GOET) | **KF556438** | **KF556198** | **KF555970** |
| *L. debilis* | Costa Rica (III), La Gamba | Schluder 7 (GOET) | **KF556437** | **KF556197** | **KF555969** |
| *L. deplanata* Nees | Dominican Rep., San José de Ocoa | Schäfer-Verwimp & Verwimp 26636 (GOET) | **KF556442** | **KF556202** | **KF555974** |
| *L. deplanata* | Ecuador (I), Imbabura | Schäfer-Verwimp et al. 24260/A (GOET) | **KF556440** | **KF556200** | **KF555972** |
| *L. deplanata* | Ecuador (II), Pichincha | Schäfer-Verwimp et al. 24252/A (GOET) | **KF556441** | **KF556201** | **KF555973** |
| *L. deplanata* | Ecuador (III), Pichincha | Schäfer-Verwimp et al. 24502/C (GOET) | **KF556439** | **KF556199** | **KF555971** |
| *L. discreta* Lindenb. | Australia | Thiers & Halling 2219 (L) | **KF556444** | **KF556206** | **KF555977** |
| *L. discreta* | China | Zhu 89038 (JE) | ----------- | **KF556204** | **KF555976** |
| *L. discreta* | Fiji Isls. | Pócs & Pócs 03289/CA (EGR) | **KF556443** | **KF556203** | **KF555975** |
| *L. discreta* | Indonesia | Gradstein 12032 (GOET) | ----------- | **KF556205** | ----------- |
| *L. drehwaldii* Heinrichs & Schäf.-Verw. | Peru | Drehwald 4384 (JE) | **KF556445** | **KF556207** | **KF555978** |
| *L. drummondii* Taylor | Australia (I) | Streimann 16698 (JE) | ----------- | ----------- | **KF555979** |
| *L. drummondii* | Australia (II) | Pamt 38AL (JE) | ----------- | **KF556208** | **KF555980** |
| *L. drummondii* | Australia (III) | Jarman 91/4 (JE) | ----------- | **KF556209** | **KF555981** |
| *L. drummondii* | Australia (IV) | Renner 872058 (NSW) | ----------- | JF308584 | JF308555 |
| *L. eckloniana* Lindenb. | Azores | Schäfer-Verwimp & Verwimp 29528 (GOET) | **KF556448** | **KF556212** | **KF555984** |
| *L. eckloniana* | La Palma | Schäfer- Verwimp 24788 (GOET) | DQ983690 | DQ987457 | DQ987357 |
| *L. eckloniana* | Madeira (I) | Stech 04-271 (L) | **KF556446** | **KF556210** | **KF555982** |
| *L. eckloniana* | Madeira (I) | Stech 04-433 (L) | **KF556447** | **KF556211** | **KF555983** |
| *L. epiphylla* Colenso | New Zealand | Renner 300056 (AK) | ----------- | JF308568 | JF308539 |
| *L. exilis* (Reinw., Blume & Nees) Grolle | Australia | Renner 872056 (NSW) | ----------- | JF308583 | JF308554 |
| *L. exilis* | Indonesia (I) | Schäfer-Verwimp & Verwimp 25231 (GOET) | **KF556449** | **KF556213** | **KF555985** |
| *L. exilis* | Indonesia (II) | Schäfer-Verwimp & Verwimp 24853 (GOET) | **KF556451** | **KF556215** | **KF555987** |
| *L. exilis* | Indonesia (III) | Schäfer-Verwimp & Verwimp 21080/B (GOET) | **KF556450** | **KF556214** | **KF555986** |
| *L. exilis* | Malaysia | Gradstein 10336 (GOET) | DQ983691 | DQ987472 | ----------- |
| *L. flava* (Sw.) Nees | Brazil (I) | Gradstein (GOET) | **KF556480** | **KF556244** | **KF556010** |
| *L. flava* | Brazil (II) | Gradstein s.n. (GOET) | DQ983692 | DQ987413 | DQ987309 |
| *L. flava* | La Palma | Schäfer-Verwimp & Verwimp 24780 (GOET) | DQ983693 | ----------- | DQ987363 |
| *L. flava* | Tenerife | Drehwald & Drehwald 4121 (GOET) | **KF556478** | **KF556242** | **KF556008** |
| *L. flava* | Dominican Rep. (I) | Schäfer-Verwimp & Verwimp 26855/B (GOET) | **KF556479** | **KF556243** | **KF556009** |
| *L. flava* | Dominican Rep. (II) | Schäfer-Verwimp & Verwimp 27043 (GOET) | **KF556452** | **KF556216** | **KF556612** |
| *L. flava* | Easter Island | Ireland & Bellolio 30111 (JE) | **KF556469** | **KF556234** | **KF556001** |
| *L. flava* | Ecuador (I) | Schäfer-Verwimp & Preussing 23435/A (GOET) | **KF556454** | **KF556218** | **KF556613** |
| *L. flava* | Ecuador (II) | Schäfer-Verwimp & Preussing 23203/B (GOET) | **KF556471** | **KF556236** | **KF556003** |
| *L. flava* | Fiji Isls. | Pócs & Pócs 03269/F (EGR) | **KF556463** | **KF556227** | **KF555996** |
| *L. flava* | Gough Island | Gremmen 2005-0794 (EGR) | **KF556484** | **KF556248** | **KF556013** |
| *L. flava* | Guadeloupe | Schäfer-Verwimp & Verwimp 22495 (GOET) | **KF556473** | **KF556237** | **KF556005** |
| *L. flava* | India | Schäfer-Verwimp & Verwimp 28396 (GOET) | **KF556462** | **KF556226** | **KF555995** |
| *L. flava* | Indonesia (I) | Schäfer-Verwimp & Verwimp 25243/C (GOET) | **KF556453** | **KF556217** | **KF555988** |
| *L. flava* | Indonesia (II) | Schäfer-Verwimp & Verwimp 21078/A (GOET) | **KF556466** | **KF556230** | **KF555998** |
| *L. flava* | Indonesia (III) | Gradstein 12037 (GOET) | **KF556481** | **KF556245** | **KF556619** |
| *L. flava* | Indonesia (IV) | Schäfer-Verwimp & Verwimp 24907 (GOET) | **KF556467** | **KF556231** | **KF556616** |
| *L. flava* | Indonesia (V) | Schäfer-Verwimp & Verwimp 24825 (GOET) | **KF556460** | **KF556224** | **KF555993** |
| *L. flava* | Indonesia (VI) | Schäfer-Verwimp & Verwimp 24806/B (GOET) | **KF556455** | **KF556219** | **KF555989** |
| *L. flava* | Indonesia (VII) | Schäfer-Verwimp & Verwimp 24901/A (GOET) | ----------- | **KF556232** | **KF555999** |
| *L. flava* | Kenya (I) | Chuah et al. 03017/H (EGR) | **KF556566** | **KF556347** | ----------- |
| *L. flava* | Kenya (II) | Pócs & Pócs 04011/AY (EGR) | ----------- | **KF556250** | **KF556620** |
| *L. flava* | Kenya (III) | Pócs & Pócs 04013/M (EGR) | **KF556586** | **KF556368** | **KF556120** |
| *L. flava* | Kenya (VI) | Pócs & Pócs 04026/X (EGR) | **KF556587** | **KF556369** | **KF556121** |
| *L. flava* | Kenya (V) | Pócs & Pócs 04026/X (EGR) | **KF556588** | **KF556370** | **KF556635** |
| *L. flava* | Madeira (I) | Drehwald 3722 (GOET) | **KF556472** | **-----------** | **KF556004** |
| *L. flava* | Madeira (II) | Stech 09-425 (L) | **KF556474** | **KF556238** | **KF556006** |
| *L. flava* | Malaysia (I) | Schäfer-Verwimp & Verwimp 18541/C (GOET) | **KF556459** | **KF556223** | **KF555992** |
| *L. flava* | Malaysia (II) | Schäfer-Verwimp & Verwimp 18861/B (GOET) | **KF556458** | **KF556222** | **KF556614** |
| *L. flava* | Malaysia (III) | Schäfer-Verwimp & Verwimp 18528/A (GOET) | **KF556461** | **KF556225** | **KF555994** |
| *L. flava* | Nepal | Long 17308 (JE) | **KF556470** | **KF556235** | **KF556002** |
| *L. flava* | New Zealand, North Island | Braggins 90/30 (GOET) | **KF556475** | **KF556239** | **KF556617** |
| *L. flava* | Panama | Schäfer-Verwimp & Verwimp 30873 (GOET) | **KF556482** | **KF556246** | **KF556011** |
| *L. flava* | Réunion (I) | Gradstein 12013 (GOET) | **KF556465** | **KF556229** | **KF555997** |
| *L. flava* | Réunion (II) | Pócs 9501/J (EGR) | **KF556487** | **KF556252** | **KF556016** |
| *L. flava* | Réunion (III) | Pócs 08068/Z (EGR) | **KF556486** | **KF556251** | **KF556015** |
| *L. flava* | São Tomé and Príncipe | Shevock 34217 (EGR) | **KF556464** | **KF556228** | **KF556615** |
| *L. flava* | South Africa | Arts RSA 25/LL (JE) | ----------- | **KF556233** | **KF556000** |
| *L. flava* | Sri Lanka (I) | Schäfer-Verwimp & Verwimp 15761 (GOET) | **KF556428** | **KF556184** | **KF555959** |
| *L. flava* | Sri Lanka (II) | Schäfer-Verwimp & Verwimp 5631 (GOET) | **KF556456** | **KF556220** | **KF555990** |
| *L. flava* | USA (I) | Majestyk & Wilbur 9979 (DUKE) | **KF556476** | **KF556240** | **KF556618** |
| *L. flava* | USA (II) | Nelson et al. 18549 (DUKE) | **KF556477** | **KF556241** | **KF556007** |
| *L. grossitexta* (Steph.) M.E.Reiner & Goda | Brazil, Paraná | Schäfer-Verwimp & Verwimp 10920 (GOET) | **KF556490** | **KF556255** | **KF556019** |
| *L. grossitexta* | Ecuador (I) | Schäfer-Verwimp & Nebel 31985 (GOET) | **KF556489** | **KF556254** | **KF556018** |
| *L. grossitexta* | Ecuador (II) | Schäfer-Verwimp & Nebel 32151/A (GOET) | **KF556488** | **KF556253** | **KF556017** |
| *L. grossitexta* | Panama | Schäfer-Verwimp & Verwimp 31000 (GOET) | **KF556491** | **KF556256** | **KF556020** |
| *L. helmsiana* Steph. | New Zealand (I) | Renner 300050 (AK) | ----------- | JF308567 | JF308538 |
| *L. helmsiana* | New Zealand (II) | Renner 300069a (AK) | ----------- | JF308570 | JF308541 |
| *L. helmsiana* | New Zealand (III) | Renner 300069 (AK) | ----------- | JF308569 | JF308540 |
| *L. hibernica* Grolle | Ireland | Long 11743 (JE) | ----------- | **KF556257** | **KF556021** |
| *L. holtii* Spruce | Madeira | Drehwald & Drehwald 3719 (GOET) | **KF556492** | **KF556258** | **KF556022** |
| *L. intricata* J.B.Jack & Steph. | Ecuador | Schäfer-Verwimp & Nebel 33217 (GOET) | ----------- | ----------- | **KF556023** |
| *L. isocalycina* (Nees) Spruce | Bolivia | Gradstein 7492 (GOET) | **KF556495** | **KF556261** | **KF556026** |
| *L. isocalycina* | Brazil (I) | Costa & Gradstein 3720 (GOET) | **KF556496** | **KF556262** | **KF556027** |
| *L. isocalycina* | Brazil (II) | Costa & Gradstein 3864 (GOET) | **KF556493** | **KF556259** | **KF556024** |
| *L. isocalycina* | Brazil (III) | Costa & Gradstein 3863 (GOET) | **KF556494** | **KF556260** | **KF556025** |
| *L. isophylla* E.W.Jones | Madagascar | Lübenau 21 (EGR) | **KF556497** | **KF556263** | **KF556028** |
| *L. japonica* Mitt. | Japan (I) | Mizutani 15618 (L) | **KF556499** | **KF556265** | **KF556030** |
| *L. japonica* | Japan (II) | Mizutani 14074 (L) | **KF556498** | **KF556264** | **KF556029** |
| *L. japonica* | Russia | Bakalin HRE 63 (GOET) | **KF556500** | **KF556266** | **KF556031** |
| *L. laeta* (Lehm. & Lindenb.) Gottsche, Lindenb. & Nees | Ecuador | Schäfer-Verwimp et al. 24412 (GOET) | **KF556501** | **KF556267** | **KF556032** |
| *L. laetevirens* Nees & Mont. | Argentina | Reiner MER 985 (JE) | ----------- | **KF556278** | **KF556040** |
| *L. laetevirens* | Bolivia | Linneo et al. 533 (MO) | **KF556505** | **KF556271** | **KF556034** |
| *L. laetevirens* | Costa Rica (I) | Schäfer-Verwimp & Holz SV/H-0315/A (GOET) | ----------- | **KF556274** | **KF556623** |
| *L. laetevirens* | Costa Rica (II) | Schäfer-Verwimp & Holz SV/H-0406 (GOET) | **KF556507** | **KF556273** | **KF556036** |
| *L. laetevirens* | Dominican Rep. (I) | Schäfer-Verwimp & Verwimp 27079 (GOET) | **KF556508** | **KF556275** | **KF556037** |
| *L. laetevirens* | Dominican Rep. (II) | Schäfer-Verwimp & Verwimp 27049 (GOET) | **KF556502** | **KF556268** | **KF556033** |
| *L. laetevirens* | Dominican Rep. (III) | Schäfer-Verwimp & Verwimp 27009/A (GOET) | **KF556503** | **KF556269** | **KF556621** |
| *L. laetevirens* | Dominican Rep. (IV) | Schäfer-Verwimp & Verwimp 27166 (GOET) | **KF556509** | **KF556276** | **KF556038** |
| *L. laetevirens* | Ecuador | Schäfer-Verwimp & Nebel 31905/A (GOET) | **KF556504** | **KF556270** | **KF556622** |
| *L. laetevirens* | French Guiana | Holz FG00-311 (GOET) | ----------- | **KF556277** | **KF556039** |
| *L. laetevirens* | Madeira (I) | Schäfer-Verwimp & Verwimp 26013A (GOET) | **KF556406** | **KF556161** | **KF555939** |
| *L. laetevirens* | Madeira (II) | Schäfer-Verwimp & Verwimp 26013B (GOET) | **KF556407** | **KF556162** | **KF555940** |
| *L. laetevirens* | Mexico | Gradstein & Velasquez s.n. (GOET) | **KF556506** | **KF556272** | **KF556035** |
| *L. lamacerina* (Steph.) Schiffn. | Azores (I) | Schäfer-Verwimp & Verwimp 29394 (GOET) | **KF556510** | **KF556279** | **KF556041** |
| *L. lamacerina* | Azores (II) | Schäfer-Verwimp & Verwimp 29325/A (GOET) | **KF556513** | **KF556282** | **KF556044** |
| *L. lamacerina* | Canada | Schofield & Schofield 95548 (DUKE) | **KF556514** | **KF556283** | **KF556045** |
| *L. lamacerina* | Ireland | Long 11625 (JE) | ----------- | **KF556286** | **KF556047** |
| *L. lamacerina* | Madeira (I) | Stech 04-297 (L) | **KF556512** | **KF556281** | **KF556043** |
| *L. lamacerina* | Madeira (II) | Stech 04-361 (L) | **KF556511** | **KF556280** | **KF556042** |
| *L. lamacerina* | USA (I) | Risk 13222 (DUKE) | **KF556515** | **KF556284** | **KF556624** |
| *L. lamacerina* | USA (II) | Shaw 10368 (DUKE) | **KF556516** | **KF556285** | **KF556046** |
| *L. lomana* E.W.Jones | Réunion (I) | Pócs 08068/P (EGR) | **KF556389** | **KF556139** | **KF556602** |
| *L. lomana* | Réunion (II) | Pócs 08064/L (EGR) | **KF556388** | **KF556138** | **KF555921** |
| *L. lumbricoides* (Nees) Nees | Indonesia | Gradstein & Ariyanti 11028 (GOET) | ----------- | **KF556287** | **KF556048** |
| *L. micholitzii* Mizut. | Fiji Isls. (I) | Pócs & Pócs 03288/DC (EGR) | **KF556517** | **KF556288** | **KF556049** |
| *L. micholitzii* | Fiji Isls. (II) | Pócs & Pócs 03309/L (EGR) | **KF556518** | **KF556289** | **KF556625** |
| *L. micholitzii* | Indonesia | Schäfer-Verwimp & Verwimp 24923/E (GOET) | **KF556519** | **KF556290** | **KF556050** |
| *L. microloba* Taylor | Fiji Isls. (I) | Pócs & Pócs 03280/CC (EGR) | ----------- | **KF556292** | **KF556626** |
| *L. microloba* | Fiji Isls. (II) | Pócs 08013/Y (EGR) | **KF556520** | **KF556291** | **KF556051** |
| *L. microloba* | Fiji Isls. (III) | Pócs & Pócs 03279/CK (EGR) | **KF556521** | **KF556293** | **KF556627** |
| *L. mimula* Hürl. | Fiji Isls. (I) | Pócs 08013/M (EGR) | **KF556522** | **KF556294** | **KF556628** |
| *L. mimula* | Fiji Isls. (II) | Pócs 08034/E (EGR) | **KF556523** | **KF556295** | **KF556052** |
| *L. mimula* | Indonesia (I) | Schäfer-Verwimp & Verwimp 16973 (GOET) | **KF556524** | **KF556296** | **KF556053** |
| *L. mimula* | Indonesia (II) | Schäfer-Verwimp 20930 (GOET) | AY548104 | DQ238580 | DQ987261 |
| *L. minutiloba* A.Evans | Easter Island | Ireland & Bellolio 30138 (JE) | **KF556525** | **KF556297** | **KF556054** |
| *L. monimiae* (Steph.) Steph. | Ecuador | Schäfer-Verwimp & Preussing 23226/A (GOET) | **KF556526** | **KF556298** | **KF556055** |
| *L. multidentata* M*.*E.Reiner & Mustelier | Dominican Rep. (I) | Pócs & Pócs 03157/A (EGR) | **KF556528** | **KF556300** | **KF556057** |
| *L. multidentata* | Dominican Rep. (II) | Pócs & Pócs 03157/A (EGR) | **KF556527** | **KF556299** | **KF556056** |
| *L. neelgherriana* Gottsche | Japan | Higuchi BSE 1295 (L) | ----------- | **KF556301** | **KF556058** |
| *L. nepalensis* (Steph.) H.A.Mill., Bonner & Bischl. | Nepal | Long 17250 (JE) | ----------- | **KF556302** | **KF556059** |
| *L. obscura* Mitt. | Malaysia | Schäfer-Verwimp & Verwimp 18745/B (GOET) | ----------- | **KF556304** | **KF556060** |
| *L. obscura* | Indonesia | Schäfer-Verwimp & Verwimp 16737 (GOET) | **KF556530** | ----------- | **KF556630** |
| *L. obtusangula* | Bolivia (I) | Gradstein 9948 (GOET) | DQ983731 | DQ987428 | DQ987324 |
| *L. obtusangula* Spruce | Bolivia (II) | Krömer 869 (GOET) | **KF556532** | **KF556307** | **KF556063** |
| *L. obtusangula* | Bolivia (III) | Krömer 1061 (GOET) | ----------- | **KF556306** | **KF556062** |
| *L. obtusangula* | French Guiana | Holz FG 00-291B (GOET) | **KF556531** | **KF556305** | **KF556061** |
| *L. oligoclada* Spruce | Brazil (I) | Schäfer-Verwimp & Verwimp 13590 (GOET) | **KF556533** | **KF556308** | **KF556064** |
| *L. oligoclada* | Brazil (II) | Schäfer-Verwimp & Verwimp 10560 (GOET) | **KF556534** | **KF556310** | ----------- |
| *L. oligoclada* | Brazil (III) | Schäfer-Verwimp & Verwimp 11780 (GOET) | ----------- | **KF556309** | ----------- |
| *L. oracola* M.A.M.Renner | New Zealand (I) | Renner 299972 (AK) | ----------- | JF308557 | JF308528 |
| *L. oracola* | New Zealand (II) | Renner 300010 (AK) | ----------- | JF308560 | JF308531 |
| *L. oracola* | New Zealand (III) | Renner 300078 (AK) | ----------- | JF308571 | JF308542 |
| *L. oracola* | New Zealand (IV) | Renner 300012 (AK) | ----------- | JF308561 | JF308532 |
| *L. oracola* | New Zealand (V) | Renner 300003 (AK) | ----------- | JF308559 | JF308530 |
| *L. osculatiana* De Not. | Costa Rica | Schäfer-Verwimp & Holz SV/H-0496/B (GOET) | **KF556539** | **KF556315** | **KF556068** |
| *L. osculatiana* | Ecuador | Schäfer-Verwimp et al. 24526 (GOET) | **KF556535** | **KF556311** | **KF556065** |
| *L. osculatiana* | Panama | Schäfer-Verwimp & Verwimp 30958 (GOET) | **KF556538** | **KF556314** | **KF556631** |
| *L. osculatiana* | Venezuela | Drehwald & Reiner-Drehwald 40081 (GOET) | **KF556537** | **KF556313** | **KF556067** |
| *L. pallescens* Mitt. | Ecuador (I) | Schäfer-Verwimp & Nebel 32731 (GOET) | **KF556540** | ----------- | **KF556069** |
| *L. pallescens* | Ecuador (II) | Schäfer-Verwimp & Nebel 31824 (GOET) | **KF556541** | **KF556316** | **KF556070** |
| *L. parva* (S.Hatt.) Mizut. | Japan (I) | Mizutani 16204 (L) | **KF556543** | **KF556319** | **KF556073** |
| *L. parva* | Japan (II) | Inoue 959 (JE) | ----------- | **KF556317** | **KF556071** |
| *L. parva* | Japan (III) | Mizutani 15293 (L) | **KF556542** | **KF556318** | **KF556072** |
| *L. patriciae*  Schäf.-Verw. | Malaysia | Schäfer-Verwimp & Verwimp 18583 (GOET) | ----------- | **KF556320** | **KF556074** |
| *L. paucidentata* (Steph.) Grolle | Cuba | Pócs & Caluff 9199/CL (JE) | ----------- | **KF556321** | **KF556075** |
| *L. paucidentata* | Dominica | Schäfer- Verwimp 17737 (GOET) | DQ983695 | ----------- | DQ987345 |
| *L. phyllobola* Nees & Mont. | Ecuador | Noeske et al. 204 (GOET) | **KF556600** | **KF556322** | **KF556076** |
| *L. phyllobola* | Kenya | Pócs & Pócs 04009/B (EGR) | **KF556544** | **KF556323** | **KF556632** |
| *L. pterigonia* (Lehm. & Lindenb.) Mont. | Bolivia (I) | Churchill et al. 21851 (GOET) | **KF556546** | **KF556325** | **KF556078** |
| *L. pterigonia* | Bolivia (II) | Gradstein 9963 (GOET) | **KF556548** | **KF556327** | **KF556080** |
| *L. pterigonia* (Lehm. & Lindenb.) Mont. | Bolivia (III) | Gradstein 9964 (GOET) | DQ983732 | DQ987429 | DQ987325 |
| *L. pterigonia* | Bolivia (IV) | Churchill et al. 23467 (GOET) | **KF556547** | **KF556326** | **KF556079** |
| *L. pterigonia* | Brazil | Costa & Gradstein 3867 (GOET) | **KF556545** | **KF556324** | **KF556077** |
| *L. pterigonia* | Ecuador | Nöske 164 (GOET) | **KF556549** | **KF556328** | **KF556081** |
| *L. puiggariana* Steph. | Dominican Rep. (I) | Schäfer-Verwimp & Verwimp 27016/A (GOET) | **KF556550** | **KF556329** | **KF556082** |
| *L. puiggariana* | Dominican Rep. (II) | Schäfer-Verwimp & Verwimp 27215/A (GOET) | **KF556551** | **KF556330** | **KF556083** |
| *L. pulverulenta* (Gottsche ex Steph.) M.E.Reiner | Bolivia | Reiner-Drehwald & Drehwald 4517 (GOET) | **KF556552** | **KF556331** | **KF556084** |
| *L. ramosissima* Steph. | São Tomé and Príncipe (I) | Shevock 34551 (EGR) | ----------- | **KF556334** | **KF556087** |
| *L. ramosissima* | São Tomé and Príncipe (II) | Shevock 34348A (EGR) | **KF556554** | **KF556333** | **KF556086** |
| *L. ramosissima* | São Tomé and Príncipe (III) | Shevock 34451 (EGR) | **KF556553** | **KF556332** | **KF556085** |
| *L. ramulosa* (Herzog) R.M.Schust. | Costa Rica | Schäfer-Verwimp & Holz SV/H-0229/A (GOET) | **KF556555** | **KF556335** | **KF556088** |
| *L. ramulosa* | Dominican Rep. | Schäfer-Verwimp & Verwimp 26954 (GOET) | ----------- | **KF556337** | **KF556633** |
| *L. ramulosa* | Ecuador (I) | Wilson et al. 04-24 (GOET) | **KF556556** | **KF556336** | **KF556089** |
| *L. ramulosa* | Ecuador (II) | Schäfer-Verwimp et al. 24208 (GOET) | **KF556557** | **KF556338** | **KF556090** |
| *L. reflexistipula* (Lehm. & Lindenb.) Gottsche, Lindenb. & Nees | Brazil | Schäfer-Verwimp & Verwimp 12482 (GOET) | **KF556558** | **KF556339** | **KF556091** |
| *L. reflexistipula* | Ecuador (I) | Schäfer-Verwimp & Nebel 31707 (GOET) | **KF556560** | **KF556341** | **KF556093** |
| *L. reflexistipula* | Ecuador (II) | Schäfer-Verwimp et al. 24215/A (GOET) | **KF556563** | **KF556344** | **KF556096** |
| *L. reflexistipula* | Ecuador (III) | Schäfer-Verwimp & Nebel 33162 (GOET) | **KF556559** | **KF556340** | **KF556092** |
| *L. reflexistipula* | Ecuador (IV) | Schäfer-Verwimp & Nebel 32032 (GOET) | **KF556561** | **KF556342** | **KF556094** |
| *L. reflexistipula* | Peru | Drehwald 10046 (GOET) | **KF556562** | **KF556343** | **KF556095** |
| *L. reflexistipula* var. *costaricensis* (Steph.) M.E.Reiner | Costa Rica | Schäfer-Verwimp & Holz SV/H-0434 (GOET) | **KF556565** | **KF556346** | **KF556098** |
| *L. reflexistipula* var. *costaricensis* | Panama | Schäfer-Verwimp & Verwimp 30930 (GOET) | **KF556564** | **KF556345** | **KF556097** |
| *L. rhigophila* M.A.M.Renner | New Zealand | Renner 300147 (AK) | ----------- | JF308579 | JF308550 |
| *L. rhigophila* | New Zealand | Renner 300044a (AK) | ----------- | JF308566 | JF308537 |
| *L. rhigophila* | New Zealand | Renner 300149 (AK) | ----------- | JF308580 | JF308551 |
| *L. rotundifolia* Mitt. | Costa Rica, Cartago | Schäfer-Verwimp & Holz SV/H-0378 (GOET) | **KF556567** | **KF556348** | **KF556099** |
| *L. rotundifolia* | Ecuador | Gradstein 10172 (GOET) | DQ983734 | DQ987410 | DQ987304 |
| *L. rotundifolia* | Panama, Chiriqui | Schäfer-Verwimp & Verwimp 31029 (GOET) | **KF556568** | **KF556349** | **KF556100** |
| *L. ruthii* (A.Evans) R.M.Schust. | USA (I), North Carolina | Duell 1411 p.p. (JE) | ----------- | **KF556350** | **KF556101** |
| *L. ruthii* | USA (II), Tennessee | Zartman 681 (DUKE) | **KF556569** | ----------- | **KF556634** |
| *L. sordida* (Nees) Nees | Fiji Isls. (I) | Pócs & Pócs 03300/AP (EGR) | **KF556570** | **KF556351** | **KF556102** |
| *L. sordida* | Fiji Isls. (II) | Pócs & Pócs 03305/J (EGR) | **KF556572** | **KF556353** | **KF556104** |
| *L. sordida* | Fiji Isls. (III) | Pócs & Pócs 03305/J (EGR) | **KF556571** | **KF556352** | **KF556103** |
| *L. sordida* | Indonesia | Sporn 101 (GOET) | **KF556574** | **KF556355** | **KF556106** |
| *L. sordida* | Japan | Yamaguchi 29848 (GOET) | **KF556575** | **KF556356** | **KF556107** |
| *L. sordida* | Papua New Guinea | Streimann 41611 (JE) | **KF556573** | **KF556354** | **KF556110** |
| *L. sporadica* Besch. & Spruce | Panama | Schäfer-Verwimp & Verwimp 31033 (GOET) | **KF556583** | ----------- | **KF556117** |
| *L. subspathulata* Spruce | Colombia | Gradstein 8991 (GOET) | **KF556584** | **KF556366** | **KF556118** |
| *L. subspathulata* | Dominica | Gradstein 6643 (GOET) | **KF556585** | **KF556367** | **KF556119** |
| *L. tapajosensis* Spruce | Ecuador | Nöske et al. 204 (GOET) | **KF556589** | **KF556371** | **KF556122** |
| *L. tasmanica* Gottsche | New Zealand | Renner 872054 (NSW) | ----------- | JF308581 | JF308552 |
| *L. topoensis* Gradst. & M.E.Reiner | Ecuador (I) | Gradstein & Jost 10163 (GOET) | **KF556590** | **KF556372** | **KF556123** |
| *L. topoensis* | Ecuador (II) | Gradstein & Jost 10063 (GOET) | DQ983712 | DQ987416 | DQ987312 |
| *L. topoensis* | Ecuador (III) | Gradstein & Jost 10063a (GOET) | **KF556591** | **KF556373** | **KF556124** |
| *L. topoensis* | Ecuador (IV) | Wilson et al 04-04 (GOET) | DQ983733 | DQ987435 | DQ987331 |
| *L. trinitensis* Lindenb. & Gottsche | Bolivia | Linneo et al. 82 (GOET) | **KF556593** | **KF556375** | **KF556126** |
| *L. trinitensis* | Brazil | Vital 10.168 (JE) | **KF556594** | **KF556376** | **KF556127** |
| *L. trinitensis* | Mayotte | Pócs & Pócs 05097/C (EGR) | **KF556592** | **KF556374** | **KF556125** |
| *L. tuberculosa* Steph. | São Tomé and Príncipe (I) | Pócs & Pócs 34690 (EGR) | **KF556595** | **KF556377** | **KF556128** |
| *L. tuberculosa* | São Tomé and Príncipe (II) | Shevock 34776 (EGR) | **KF556483** | **KF556247** | **KF556012** |
| *L. tuberculosa* | Thailand | Schäfer-Verwimp & Verwimp 23880 (GOET) | **KF556457** | **KF556221** | **KF555991** |
| *L. tumida* Mitt. | New Zealand (I) | Renner 299949 (AK) | ----------- | JF308556 | JF308527 |
| *L. tumida* | New Zealand (II) | Renner 300002 (AK) | ----------- | JF308558 | JF308529 |
| *L. umbilicata* (Nees) Nees et al. | Indonesia (I) | Gradstein 12076 (GOET) | **KF556598** | **KF556380** | **KF556131** |
| *L. umbilicata* | Indonesia (II) | Gradstein 51 (GOET) | **KF556599** | **KF556381** | **KF556132** |
| *L. umbilicata* | Indonesia (III) | Schäfer-Verwimp & Verwimp 20794/B (GOET) | **KF556596** | **KF556378** | **KF556129** |
| *L. umbilicata* | Indonesia (IV) | Schäfer-Verwimp & Verwimp 16954 (GOET) | **KF556597** | **KF556379** | **KF556130** |
| *L. wallichiana* (Lehm.) Gottsche, Lindenb. & Nees | Nepal | Long 16716 (JE) | ----------- | **KF556382** | ----------- |
| *L.* sp. I | Ecuador | Schäfer-Verwimp & Preussing 23533 (GOET) | **KF556536** | **KF556312** | **KF556066** |
| *L.* sp. II | Bolivia | Fuentes & Aldana 6473 (GOET) | **KF556429** | **KF556185** | **KF555960** |
| *L.* sp. III | Gough Island | Gremmen 2000-0075 (EGR) | **KF556579** | **KF556361** | **KF556112** |
| *L.* sp. IV | Fiji Isls. | Pócs 08029/O (EGR) | **KF556580** | **KF556362** | **KF556113** |
| *L.* sp. V | Madagascar | Geissler 3498 (EGR) | **KF556578** | **KF556360** | **KF556111** |
| *L.* sp. VI | Panama | Schäfer-Verwimp & Verwimp 30834 (GOET) | **KF556582** | **KF556365** | **KF556116** |
| *L.* sp. VII | Nepal | Pölt H3071 (JE) | ----------- | **KF556363** | **KF556114** |
| *L.* sp. VIII | Ethiopia | Hylander 5564 (EGR) | **KF556576** | **KF556357** | **KF556108** |
| *L.* sp. IX | São Tomé and Príncipe | Shevock et al. 34316 (EGR) | **KF556485** | **KF556249** | **KF556014** |
| *L.* sp. X | Madagascar | Pócs et al. 90100/C (JE) | **KF556581** | **KF556364** | **KF556115** |
| *Lepidolejeunea bidentula* (Steph.) R.M.Schust. | China | Koponen et al. 51525 (H) | AY125936 | AY144476 | AY125340 |
| *Lep. eluta* (Nees) R.M.Schust. | Bolivia (I) | Churchill & Vasquez 2180 (GOET) | AY548066 | DQ238579 | DQ987266 |
| *Lep. eluta* | Bolivia (II) | Drehwald 4833 (GOET) | DQ983696 | DQ987379 | DQ987257 |
| *Lep. integristipula* (J.B.Jack & Steph.) R.M.Schust. | Fiji Isls. | Pócs 03307/AC (GOET) | DQ983697 | DQ987417 | DQ987313 |
| *Microlejeunea. africana* Steph. | Madagascar | Lübenau 2 (JE) | KC313149 | KC313189 | KC313111 |
| *M. africana* | São Tomé and Príncipe (I) | Shevock 34576A (GOET) | KC313150 | KC313190 | KC313112 |
| *M. africana* | São Tomé and Príncipe (II) | Shevock 34576B (GOET) | KC313151 | KC313191 | KC313113 |
| *M. capillaris* (Gottsche) Steph. | Costa Rica | Schäfer-Verwimp & Holz SV/H-0489/B (JE) | KC313152 | KC313192 | KC313114 |
| *M. colombiana* Bischl. | Dominican Rep. | Schäfer-Verwimp & Verwimp 26614/A (JE) | KC313153 | KC313193 | KC313079 |
| *M. filicuspis* (Steph.) Heinrichs, Schäf.-Verw., Pócs & S.Dong | Fiji Isls. (I) | Pócs & Pócs 03306/R (EGR) | KC313137 | KC313177 | KC313099 |
| *M. filicuspis* | Fiji Isls. (II) | Pócs & Pócs 03304/A (EGR) | KC313138 | KC313178 | KC313100 |
| *M. filicuspis* | Thailand | Pócs & Pócs 07006/A (EGR) | KC313139 | KC313179 | KC313101 |
| *M. fischeri* (Tixier) Heinrichs, Schäf.-Verw., Pócs & S.Dong | Uganda (I) | Pócs & Lye 97141/T (EGR) | KC313140 | KC313180 | KC313102 |
| *M. fischeri* | Uganda (II) | Pócs & Lye 97142/AM (EGR) | KC313141 | KC313181 | KC313103 |
| *M. fischeri* | Uganda (III) | Pócs & Lye 97142/BB (EGR) | KC313142 | KC313182 | KC313104 |
| *M. fischeri* | Uganda (IV) | Pócs & Lye 97142/BQ (EGR) | KC313143 | KC313183 | KC313105 |
| *M. latitans* (Hook.f. & Taylor) Heinrichs, Schäf.-Verw., Pócs & S.Dong | New Zealand | Schäfer-Verwimp & Verwimp 13869 (JE) | KC313146 | KC313186 | KC313108 |
| *M.* sp. | Thailand | Schäfer-Verwimp & Verwimp 16293 (GOET) | ----------- | KC313196 | KC313117 |
| *M. squarrosa* (Steph.), Heinrichs, Schäf.-Verw., Pócs & S.Dong | Brazil (I) | Schäfer-Verwimp & Verwimp 14780 (JE) | KC313157 | KC313197 | KC313118 |
| *M. squarrosa* | Brazil (II) | Schäfer-Verwimp & Verwimp 14638 (JE) | KC313158 | KC313198 | KC313119 |
| *M. squarrosa* | Brazil (III) | Schäfer-Verwimp & Verwimp 13376 (GOET) | DQ983720 | DQ987446 | DQ987344 |
| *M. ulicina* (Taylor) Steph. | La Palma (I) | Schäfer-Verwimp & Verwimp 24800 (GOET) | KC313154 | KC313194 | KC313115 |
| *M. ulicina* | La Palma (II) | Schäfer-Verwimp & Verwimp 24666 (GOET) | KC313155 | KC313195 | KC313116 |
